# Cortical Regions Activated by Spectrally Degraded Speech in Adults With Single Sided Deafness or Bilateral Normal Hearing

**DOI:** 10.3389/fnins.2021.618326

**Published:** 2021-04-07

**Authors:** Harold Burton, Ruth M. Reeder, Tim Holden, Alvin Agato, Jill B. Firszt

**Affiliations:** ^1^Department of Neuroscience, Washington University School of Medicine, Saint Louis, MO, United States; ^2^Department of Otolaryngology-Head and Neck Surgery, Washington University School of Medicine, Saint Louis, MO, United States

**Keywords:** single sided deafness, normal hearing, spectrally degraded speech, functional magnetic resonance imaging, semantic processing

## Abstract

Those with profound sensorineural hearing loss from single sided deafness (SSD) generally experience greater cognitive effort and fatigue in adverse sound environments. We studied cases with right ear, SSD compared to normal hearing (NH) individuals. SSD cases were significantly less correct in naming last words in spectrally degraded 8- and 16-band vocoded sentences, despite high semantic predictability. Group differences were not significant for less intelligible 4-band sentences, irrespective of predictability. SSD also had diminished BOLD percent signal changes to these same sentences in left hemisphere (LH) cortical regions of early auditory, association auditory, inferior frontal, premotor, inferior parietal, dorsolateral prefrontal, posterior cingulate, temporal-parietal-occipital junction, and posterior opercular. Cortical regions with lower amplitude responses in SSD than NH were mostly components of a LH language network, previously noted as concerned with speech recognition. Recorded BOLD signal magnitudes were averages from all vertices within predefined parcels from these cortex regions. Parcels from different regions in SSD showed significantly larger signal magnitudes to sentences of greater intelligibility (e.g., 8- or 16- vs. 4-band) in all except early auditory and posterior cingulate cortex. Significantly lower response magnitudes occurred in SSD than NH in regions prior studies found responsible for phonetics and phonology of speech, cognitive extraction of meaning, controlled retrieval of word meaning, and semantics. The findings suggested reduced activation of a LH fronto-temporo-parietal network in SSD contributed to difficulty processing speech for word meaning and sentence semantics. Effortful listening experienced by SSD might reflect diminished activation to degraded speech in the affected LH language network parcels. SSD showed no compensatory activity in matched right hemisphere parcels.

## Introduction

Adults and children listening with single-sided deafness (SSD) suffer reduced speech recognition from degraded signal segregation and impaired communication (Firszt et al., 2017). Especially noisy environments, such as multi-talker babble, impair word identification in SSD (Bronkhorst and Plomp, 1988; Wie et al., 2010; Mattys et al., 2012; Rothpletz et al., 2012; Firszt et al., 2017). Increased speech recognition difficulties arise with SSD from lexical uncertainty due to missed or unrecognized words (Mattys et al., 2012). Adverse sound environments particularly provoked greater effort, cognitive loads, fatigue, and diminished quality of life in SSD individuals (Pichora-Fuller et al., 2016).

Low-level auditory cortex regions reorganize in individuals with SSD with increased activation to acoustic stimuli ipsilateral to the intact ear (Scheffler et al., 1998; Bilecen et al., 2000; Tschopp et al., 2000; Ponton et al., 2001; Langers et al., 2005; Hanss et al., 2009; Burton et al., 2012). With normal hearing (NH), acoustic stimulation of one ear predominantly activates contralateral auditory cortex (Zatorre and Binder, 2000), leading to right ear input dominance in LH language network regions for speech. Right ear SSD adults might use left ear ipsilateral activation of LH regions to compensate during speech recognition. Prior evidence supporting possible compensatory RH activity was of enhanced domains for non-speech sounds in early auditory cortex (EAC) for intact, hearing ear inputs in SSD adults (Burton et al., 2012). However, right ear SSD individuals did not show symmetrical hemispheric distributions of auditory evoked potentials to non-speech sounds (Hanss et al., 2009), indicating likely ineffective activation of LH language regions.

A fronto-temporo-parietal network in NH processes words and sentences in context and in relation to modulation by acoustic input (Obleser et al., 2007a; Binder et al., 2009; Binder and Desai, 2011; Turken and Dronkers, 2012; Wild et al., 2012; Zekveld et al., 2012; Seghier, 2013). Multiple cortical regions categorized in NH adults contribute to aspects of speech recognition (Davis and Johnsrude, 2003; Okada et al., 2010; Peelle et al., 2010). For example, tasks requiring semantic retrieval of less intelligible speech engage left ventral inferior frontal cortex (LIFC) and anterior parts of the angular gyrus in parietal cortex (i.e., PGi) (Obleser et al., 2007a; Peelle et al., 2010). Additionally, NH previously showed greater activation with unfavorable ambient noise in regions involved with cognitive control of working memory for speech and directed attention (Hannemann et al., 2007; Herrmann et al., 2012).

Single sided deafness might instigate activity changes beyond lower auditory cortex, especially during challenged listening to speech in noisy environments. SSD compared to NH individuals likely activate lower amplitude responses in language network regions. Hypothetically, speech recognition deficits in SSD may reflect diminished activity in one or more language network regions, suggesting possible processes deficient in SSD individuals.

Speech recognition normally progresses in stages, mostly governed by LH regions (Davis and Johnsrude, 2003; Spitsyna et al., 2006; Obleser et al., 2007a; Zatorre and Gandour, 2008 #62; Binder et al., 2009; Mattys et al., 2012). Bilateral EAC initiates processing of speech acoustics (Griffiths and Warren, 2002; Warren et al., 2005; Obleser et al., 2008; Warrier et al., 2009; Woods et al., 2011; Kyong et al., 2014; Plack et al., 2014). Speech acoustics specifically includes temporal and spectral features of phonemes and morphemes (Griffiths and Warren, 2002; Davis and Johnsrude, 2003). In SSD, deficient processing of speech acoustics in EAC might be involved.

Accessory auditory cortex (AAC) receives information about phonemes and morphemes from EAC. AAC principally processes phonetics and phonology of syllables and words (Scott and Johnsrude, 2003; Wong et al., 2008; Liebenthal et al., 2010; Okada et al., 2010; Turkeltaub and Branch Coslett, 2010; Woods et al., 2011; Specht, 2014). Similarly, sentence level processing of phonetics occurs in adjoining anterior-middle and middle-posterior temporal regions (Scott et al., 2006; Turkeltaub and Branch Coslett, 2010; Evans et al., 2014; Kyong et al., 2014). Depressed LH EAC activation in SSD might weaken phonetic and phonological processing in AAC and adjoining temporal cortex. A likely effect is deficient processing of sentence level intelligibility in SSD individuals. Thus, impaired accuracy by SSD individuals in recognizing speech might arise from inadequate activation in parts of temporal cortex lateral, posterior and anterior to EAC.

Accessory auditory cortex and neighboring temporal cortex project to left inferior frontal, inferior parietal, and indirectly, premotor cortex (Narain et al., 2003; Scott, 2008; Peelle et al., 2010). A hypothesized dedicated LH semantic language network (Binder et al., 2009) includes these regions and anterior temporal pole and posterior/middle temporal cortex (Spitsyna et al., 2006). Processing semantics and word relatedness in these regions extracts lexical and categorical information of speech (Obleser et al., 2007b; Turkeltaub and Branch Coslett, 2010; Peelle, 2012; Specht, 2014). Left inferior frontal cortex (LIFC) and lexicality processing in middle temporal cortex link to angular gyral (AG) cortex through functional and anatomical connectivity (Obleser et al., 2007a; Wang et al., 2017). Connections with PGi in AG possibly further contribute multisensory information to semantic meaning (Obleser et al., 2007a; Seghier, 2013; Xu et al., 2016).

Crucial to understanding speech are intelligibility and retrieved word meanings from a stored lexicon (Badre and Wagner, 2002; Badre et al., 2005). Previously, components of the semantic language network showed lower response amplitudes in NH to less intelligible speech from noise or spectral degradation (Davis and Johnsrude, 2007; Obleser et al., 2007b; Sharp et al., 2010; Mcgettigan et al., 2012; Wild et al., 2012; Golestani et al., 2013; Hervais-Adelman et al., 2014; Specht, 2014). Less intelligible speech, for example, activated smaller response amplitudes in LIFC of NH (Davis and Johnsrude, 2003, 2007; Obleser et al., 2007b; Mcgettigan et al., 2012; Wild et al., 2012; Golestani et al., 2013; Hervais-Adelman et al., 2014; Specht, 2014). Previously, less activation corresponded to reduced semantic retrieval and, hence lexical content (Badre and Wagner, 2002; Badre et al., 2005). The dynamic range of stimulus induced intelligibility effects may be smaller for individuals with SSD, hence leading to a reduced capacity to hear clear speech as intelligible. Individuals with SSD also might be unable or ineffectual in integrating speech with multisensory or cognitive processes from reduced activity in PGi, further resulting in an impaired linkage with a language network for intelligible speech (Obleser et al., 2007a).

Normal hearing individuals needed greater attention to speech intelligibility with decrements in SNR, hence effortful listening (Binder et al., 2009; Zekveld et al., 2011; Wild et al., 2012). In SSD cases, smaller decrements in SNR might instigate comparable declines in intelligibility and changes in cortical activity. Processing external as opposed to self-reverential events reduces metabolic activity in the default mode network (DMN) (Fox et al., 2005; Buckner et al., 2008, 2009). Normally, processing external events triggers a greater decrease from baseline metabolic load, expressed as negative blood oxygen level-dependent (BOLD) amplitudes in DMN (Raichle and Snyder, 2007). Greater effort to process speech by individuals with SSD might therefore lead to deeper negative BOLD amplitudes in the posterior cingulate cortex, part of the DMN.

Another aspect of effortful listening involves retrospective review of recently heard speech through working memory (Mattys et al., 2012). Adults with SSD may retrospectively try retrieval and review of poorly heard speech. Dorsolateral prefrontal cortex (DLPFC), essential for working memory (Kim, 2010; Petrides, 2016), might show enhanced activity in DLPFC of SSD from greater effort to recall speech.

We sought a better understanding of SSD consequences on speech evoked activity by examining activations in previously identified subdivisions (parcels) within fronto-temporo-parietal language network regions. The definition of each parcel was from multi-modal characteristics (Glasser et al., 2016). An example modal feature was task based functional activation (Barch et al., 2013). The current goal was to compare performance accuracy and cortical activation differences in adults with SSD and NH when processing spoken sentences of varied intelligibility, using three levels of spectral degradation, and of predictable or unpredictable semantics. We assessed group differences in cortical activity analyzed per previously identified parcels.

## Materials and Methods

### Participants

Each group had twelve participants. Those with right ear SSD ranged between 23–64 years and five were female; NH participants ranged between 25–66 years and seven were female. All participants were native English speakers and scored a right-hand preference on the Edinburgh Handedness Inventory (Oldfield, 1971). Left-handedness was an exclusion criterion. Participants gave informed consent following guidelines approved by the Human Research Protection Office at Washington University School of Medicine and following the Code of Ethics of the World Medical Association (Declaration of Helsinki). Recruitment of adults with SSD was through outpatient audiology and otology clinics and of NHs through Volunteer for Health program at Washington University School of Medicine.

Single sided deafness participants varied in age and etiology of hearing loss (Table 1). Two lost hearing at birth, one in early childhood and nine as adults.

**TABLE 1:** Demographics and pure tone average thresholds for SSD and NH participants.

Partic #	Gender	Age at Test	RE PTA (5,1,2)^*a*^	RE PTA (3,4,6)^*b*^	LE PTA (5,1,2)^*a*^	LE PTA (3,4,6)^*b*^	AAO SPHL^*c*^ (yrs)	DOD^*d*^ (yrs)	Etiology
**SSD**									
Spin 05	M	62	118.3	118.3	16.7	40.0	33	29	AN^*e*^
Spin 06	M	58	118.3	115.0	8.3	30.0	48	10	AN
Spin 10	F	23	105.0	112.5	0.0	–5.0	0	23	CND
Spin 11	M	64	118.3	116.7	15.0	55.0	49	15	Trauma
Spin 15	M	30	120.0	123.3	6.7	6.7	23	7	Ototoxicity
Spin 16	F	59	115.0	123.3	6.7	10.0	51	8	AN
Spin 17	M	55	118.3	116.7	8.3	21.7	6	49	Mumps
Spin 20	F	44	91.7	83.3	20.0	16.7	41	3	Unknown
Spin 21	M	50	118.3	115.0	15.0	15.0	41	9	AN
Spin 24	F	25	108.3	115.0	21.7	30.0	0	25	Unknown
Spin 25	M	37	86.7	101.7	3.3	15.0	30	7	Trauma
Spin 27	F	60	118.3	116.7	11.7	25.0	50	10	Unknown
	Mean	47.3	111.4	113.1	11.1	21.7	30.8	16.3	
	SD	15.0	11.4	10.9	6.7	15.9	19.3	13.2	
**NH**									
Spin 04	M	54	5.0	8.3	3.3	8.3			
Spin 07	F	25	0.0	0.0	3.3	5.0			
Spin 08	F	57	21.7	8.3	20.0	11.7			
Spin 09	M	63	3.3	10.0	6.7	18.3			
Spin 12	F	53	6.7	11.7	8.3	20.0			
Spin 13	M	35	15.0	11.7	15.0	15.0			
Spin 14	M	66	6.7	23.3	8.3	45.0			
Spin 18	F	57	10.0	13.3	8.3	15.0			
Spin 23	F	29	8.3	11.7	6.7	5.0			
Spin 26	M	31	10.0	8.3	10.0	11.7			
Spin 29	F	62	21.7	21.7	16.7	20.0			
Spin 28	F	46	13.3	13.3	10.0	8.3			
	Mean	48.2	10.1	11.8	9.7	15.3			
	SD	14.5	6.8	6.1	5.1	10.8			

*^*a*^PTA (5,1,2), pure tone average thresholds at 500, 1000, and 2000 Hz.*

*^*b*^PTA (3,4,6), pure tone average thresholds at 3000, 4000, and 6000 Hz.*

*^*c*^AAO SPHL, age at onset of profound hearing loss.*

*^*d*^DOD, duration of deafness.*

*^*e*^AN, acoustic neuroma.*

Hearing loss duration averaged 16.3 years and spanned from 3 to 49 years. Average age at hearing loss onset was 30.8 years. Right ear average thresholds for three low frequency pure tones (LPTA: 0.5, 1 and 2 kHz) was 111.4 dB HL (SD 11.4; range 86.7–118.3). Right ear average threshold for high frequency pure tones (HPTA: 3, 4, and 6 kHz) was 113.1 dB HL (SD 10.9; range 83.3–123.3). Left ear LPTA and HPTA thresholds in SSD were normal (LPTA: 11.1 dB HL; SD 6.7 and HPTA: 21.7 dB HL; SD 15.9) and matched NH participant PTAs in both ears (Table 1).

### Sentence Intelligibility and Predictability

Participants heard sentences from the Revised Speech Intelligibility in Noise test (R-SPIN) in which contextual information of speech affects semantic predictability (Kalikow et al., 1977; Bilger et al., 1984; Wilson and Mcardle, 2012). Recordings were of English sentences spoken by a single male talker (Wilson and Mcardle, 2012). R-SPIN uses a sentence paradigm in which a noun as the last word in the sentence is the target. There are two types of sentences: (1) high-predictability (HP) sentences supply syntactic, semantic, and prosodic cues predictive of the target word; and (2) low-predictability (LP) sentences, which supply few, if any, cues in the sentence that help predict the target word. An HP/LP pair of different sentences had the same target noun. Target words across lists had equivalent phonetic content, intelligibility, lexical frequency of occurrence, length of syllables and neighborhood density (Clarke, 2000). An example HP sentence was “Raise the flag up the POLE.” An example LP sentence with the same target was “Bob could consider the POLE” (Wilson and Mcardle, 2012). R-SPIN consists of eight 50-sentence lists (available in Wilson and Mcardle, 2012), divided equally into 25 HP and LP sentences.

There was an equal division of four 50 sentence lists into 25 HP and LP pairs of different sentences and each predictability pair ending in the same target word. Selected sentences had a mean duration of 1.78 s with a range from 1.09 to 2.49 s. A channel noise vocoder MATLAB code processed sentences into 4, 8, and 16 logarithmic spaced frequency bands with the RMS power output level matched to the channel input level. Spectral degradation (Shannon et al., 1995) parametrically reduced audible details and intelligibility of the original R-SPIN sentences. The amplitude envelop for each band was extracted and smoothed. Subsequent application of “wide-band noise in each frequency range… modulated using this amplitude envelope (…) and combined to produce a noise-vocoded sentence,” (p.224, Figure 1; Davis et al., 2005) while preserving temporal information (Davis and Johnsrude, 2003; Davis et al., 2005; Obleser and Kotz, 2010). Scott and colleagues previously found 4-, 8-, and 16-band vocoding resulted in, respectively, ∼30, 80 and 100% correct identification of sentence words (Scott et al., 2006). Thus, speech intelligibility varied directly with number of frequency bands. Spectral detail and intelligibility of the sentences were least with 4-bands, somewhat intelligible with 8-bands, and like undegraded sentences with 16-bands (Obleser et al., 2007a). The Matlab code matched output to input levels and adjusted loudness levels to equal those of unprocessed sentences (Shannon et al., 1995; Hall et al., 2000). There were six conditions based on vocoded sentences of 4-, 8-, and 16-bands creating three levels of speech intelligibility, and two levels of predictability, reflecting the semantic and syntax of sentences. The imaging study included a seventh condition of trials of silence with no sentence presentation.

**FIGURE 1:**
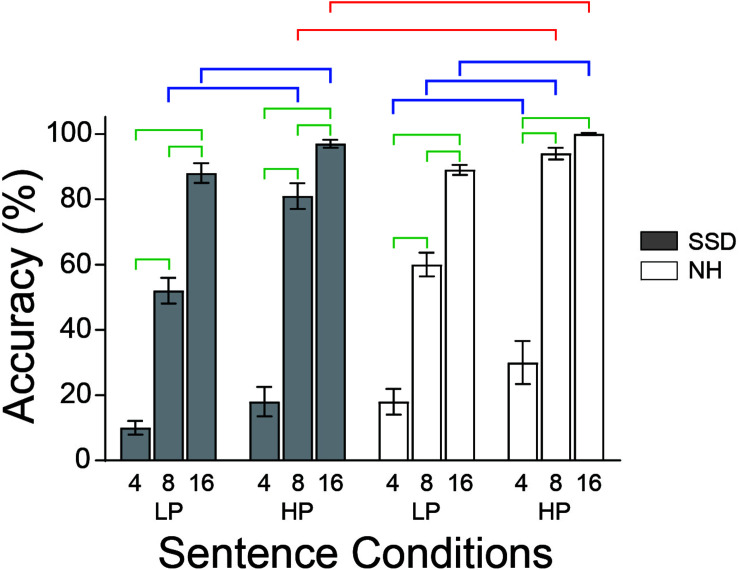
The behavioral study found average accuracies naming sentence last words varied with intelligibility and semantic predictabilities in both groups. Brackets mark bars with significant accuracy differences between intelligibility levels (green), groups (red), and predictabilities (blue). LP, low predictability; HP, high predictability; NH (white bars), normal hearing; SSD (gray bars), single sided deafness. Error bars are SEM.

There was random choice of 10 target words from the sentence pool for a given MRI run and for each of the vocoded conditions. Selection was random for the 10 target words chosen to be one of 5 LP or HP sentences for the first noise vocoded frequency band condition block in a run. Predictability condition reversed for that target word in the second presentation. Consequently, each of the target words occurred twice within the same run for the same frequency band condition but separately for LP and HP sentences and in different blocks.

For the behavioral study we assessed accuracies by participants in both groups in naming sentence last words. Participants heard R-SPIN sentences at 60dB SPL through insert earphones (EAR Tone 3a) from a clinical audiometer (GSI 61) while sitting in the middle of a large, double-walled sound booth (IAC, Model 1204A). Custom software on a PC (Dell Optiplex 960) controlled sentence presentation and sound level from a 24-bit sound card (Lynx Studio Technology L22). A professional service annually calibrated the GSI 61 audiometer.

The same participants heard sentences during MR imaging. A Mac computer presented sentences through MR compatible Sensimetrics S14 ear tip earphones, connected to an audio mixer and amplifier. Loudness was set to 75 dB(C) SPL using a speech shaped noise stimulus; RMS matched to the level of the SPIN sentences. Prior to testing, we calibrated sound intensity using a hand-held Type 2 sound level meter, fitted with a custom made 2cc acoustic coupler that formed a closed field when plugged into the sound tube of the S14 earphones.

Sentence presentation procedures differed for the imaging study. Prior to scanning but while in the scanner, participants first heard practice sentences without spectral degradation in the presence of previously recorded MRI scanner noise. Practice trials included an added 20 sentences with target nouns used only for training. Scanning sessions had 5 runs. Five added, discarded first frames compensated for magnetization equalization at the beginning of every run. Each run also presented Silence trials. Two of these trials were before presenting the first sentence, 5 were individually between each of six sentence blocks, and 3 were after the last sentence. Each run contained 6 stimulus blocks of 10 trials. Each stimulus trial presented one sentence. All trials within a given stimulus block were at a fixed vocoding level (e.g., 8-band vocoding). Block order was randomized subject to two constraints: (1) first and final sets of 3 blocks in a run had a block from each of the 3 vocoding levels; (2) two successive blocks did not have the same vocoding level (i.e., blocks 3 and 4 could not have the same vocoding level). Trials within each block had randomly assigned predictability levels and equal number of trials at each predictability level (i.e., 5 HP and 5 LP trials per block). R-SPIN sentence HP and LP pairs with a common target word randomly occurred in a run with a given vocoding level. Within a run, blocks of a given vocoding level separated these pairs. That is, for each run, for each pair of blocks at a given vocoding level, sentences had the same 10 target words but in different predictability contexts. All but 3 participants completed all 5 runs. The remaining participants completed 3 or 4 runs. Following a sentence presentation, a displayed 1 s cue prompted a behavioral response followed by a 2 s interval before the next volume acquisition (Supplementary Figure 1 illustrates trial event structure). The visual cue prompted participants to press one of two keys marking yes/no whether they predicted the target noun. On Silence trials, participants distributed key presses equally between the two response keys.

### Image Acquisition and Processing

A Siemens 3 Tesla TRIO scanner (Erlangen, Germany) with a twelve-element RF head matrix coil recorded whole brain images. Gradient recalled sequences of echo planar images (EPI) detected BOLD responses. Echo time for EPI was 27ms with a flip angle of 90°. Collected images of BOLD responses were from 33 contiguous, axial slices aligned parallel to a plane defined by the anterior-posterior commissures. Image collection was across interleaved odd-even numbered slices with no gap. Resolution within each 4 mm slice was 4 mm × 4 mm (64 × 64, FOV = 256 mm × 256 mm). Whole brain volume acquisition of images occurred during five runs per participant. Volume acquisition (TA) of brain images was during the first 2 s of each repetition time (TR) of 9 s (Supplementary Figure 1 illustrates trial event timing).

Participants heard a sentence during 7 s with no scanner noise per brain imaging frame (Supplementary Figure 1), resulting in a sparse temporal sampling sequence (Hall et al., 1999). Audible midpoints of each sentence occurred at 4 s prior to the onset of the next frame (see timing tick marks in Supplementary Figure 1). BOLD response peaks activated from mid-sentence coincided with the 2 s of volume acquisition at the beginning of a subsequent frame, based on 5 to 6 s hemodynamic response function (HRF) delays (Boynton et al., 1996). BOLD for the target word for sentence durations well beyond a mean of 1.78 s possibly occurred post TAs. On average, the timing might have missed, on average, a BOLD evoked by a target word but not the prior words in a sentence. The HRF consistently captured BOLD across stimulus conditions, suggesting only an inefficient design for target words but not the critical semantics of sentences. Participants had to attend the prior sentence in anticipation of the target word, resulting in a consequent affected HRF likely peaking during the TA. Timing for sentence presentations and later image analyses relied on sequentially saved times for successive frames from a scan session. Delays separated cues to respond and key presses from the ends of sentences. BOLD responses activated by the cue and key press diminished in the 9 s before the following frame, and thus were separable from BOLD activation to a sentence.

Two magnetization prepared rapid gradient echo (MP-RAGE) T1-weighted sequences captured brain structural images. Whole brain volumes in each MP-RAGE included 176 sagittal slices with a resolution of 1 mm × 1 mm × 1.25 mm. Sequence parameters included frame time of 2100 ms (TR), echo time of 3.93 ms (TE), inversion time of 1000 ms (TI) and flip angle of 7°. Software converted original MP-RAGE DICOM to NIFTI format to obtain initial high-resolution brain structural images^1^. A brain extraction program (FSL^2^) separated the brain from non-brain structures uniquely for each participant. FSL software subsequently co-registered functional EPI imaging data to the extracted brain from each participant after re-sampling to 2 mm resolution.

We used FSL FEAT (FMRI Expert Analysis Tool, Version 5.91) to perform a first level, fixed-effects analysis to process images from each functional imaging run per participant. Processing included motion correction (MCFLIRT; Motion Correction FMRIB’s Linear Registration Tool), slice-timing correction, and spatial smoothing with a 4 mm full-width half-maximum Gaussian kernel. Pre-whitening dealt with temporal autocorrelation to improve estimation efficiency for each time series per voxel. FSL analysis named sentence conditions as original explanatory variables in the design matrix (e.g., 4HP, 4LP, 8HP, 8LP, 16HP, and 16LP). Contrasts were between BOLD signals for each sentence condition compared to an average of BOLD present during Silence trials across runs. In all but two cases the average was for 50 Silence trials. FSL’s 3-column format option processed volume acquisitions immediately following each sentence condition, based on onset times identified in custom scripts for every run in each participant. A double-gamma hemodynamic response function convolved with the sentence presentation waveform to model the change in BOLD signal. A high pass temporal 90 Hz threshold filtered the images prior to computing a generalized linear model (GLM) using FILM (*FMRIB’s Improved Linear Model*) analysis, resulting in parameter estimate (PE) images for each explanatory variable. FSL software converted each PE image to a t-statistic image, then transformed into a Z-statistic image. Linear registration mapped the z-scores to a high-resolution brain for each participant. A non-linear algorithm (FNIRT: FNRIB’s Non-linear Image Registration Tool) registered these same data to the standard space of a MNI brain atlas, up-sampled to 2 mm resolution (Montreal Neurological Institute standard-space T1-weighted, average structural template image from 152 structural brain images).

A multi-step analysis co-registered results, based on segmenting and creating surface meshes of the cortex, registered to the MNI template in each participant. Generation of individualized cortical surfaces relied on an average of two T1-weighted images in volumetric space. FreeSurfer v5 (FS) software (Dale et al., 1999; Fischl et al., 2004; Ségonne et al., 2004) used the T1 images to create cortical surface reconstructions for each case. The default processing by FS generated anatomical surfaces images in volumetric space from each participant, which also deleted extraneous non-brain structures and segmented each brain into pial, cortex gray-matter mid-thickness and white matter. Subsequent manual editing improved the rendered cortical surface. Later processing created white matter and pial surface meshes, inflated surfaces to a sphere, and performed surface shape-based spherical registration of the FS surface per case to a group averaged (fsaverage) surface. A Caret operations script^3^ performed an affine transform of surface coordinates and converted these to Caret accessible metric/gifti format (Van Essen et al., 2001). The same script also registered the cortical surface mesh from each participant brain to a 164K_fs_LR (left and right hemispheres, LR) mesh, using a Conte69 human surface-based atlas for registration to the LR surface mesh (Van Essen et al., 2011).

The same Caret script converted volume images of percent signal changes to the Conte69 164k_fs_LR gifti surface files, aligning volume-based z-scores and percent signal changes to the 164K mesh. These percent signal changes were of estimates for each of the six sentence conditions from every run per participant, using procedures described in Guide for Calculating Percent Change with Featquery^4^.

Next, co-registration of average percent signal changes per participant was to previously identified and cross-validated cortical surface parcels (Glasser and Van Essen, 2011; Van Essen et al., 2011; Glasser et al., 2016) using the Conte69 brain surface. Previously established multiple anatomical, functional resting state, and task activated features identified each cortical parcel, which were located in 22 identified cortical regions [Supplementary Neuroanatomical Results in Glasser et al. (2016)].

**FIGURE 2:**
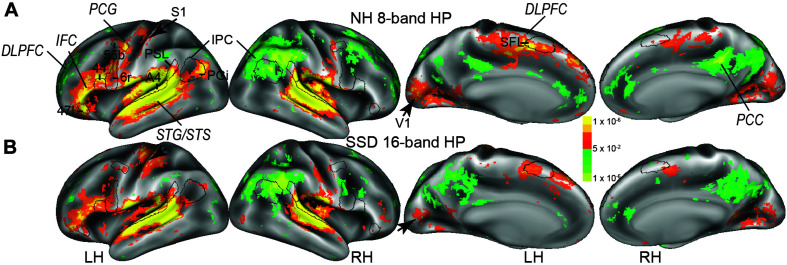
The imaging study found bilateral cluster distributions of positive and negative BOLD. **(A)** Results obtained from normal hearing (NH) to 8-band sentences with predictable semantics. **(B)** Results obtained from single sided deafness (SSD) to 16-band sentences with predictable semantics. Cortex colored red-orange to bright yellow or blue to light green had, respectively, positive and negative BOLD signal changes. Black borders encircle a few selected parcels: DLPFC, dorsolateral prefrontal cortex; IFC, inferior frontal cortex; IPC, inferior parietal cortex; PCC, posterior cingulate cortex; PCG, precentral gyrus; STG/STS, superior temporal gyrus/sulcus. Parcels: A4, 44, 47l, 55b, 6r, PGi, PSL (perisylvian language parcel), SFL (superior frontal language), S1 (primary somatosensory cortex), V1 (primary visual cortex). LH, RH left and right hemispheres.

We selected parcels for statistical analyses from 9 cortical regions encompassing cortex activated by the sentence task (Figure 2). The analyzed dependent variable was percent signal changes registered to all surface vertices enclosed within the borders of independently defined parcels (Kriegeskorte et al., 2009). A Matlab script computed average percent signal change per run, sentence condition, and participant from all cortical surface vertices within the borders of each selected LH parcel. Separately, the same script computed average percent signal changes in identically matched to RH parcels. Statistical analyses included results from trials in each run per sentence condition and from five imaging runs per participant.

### Statistical Analyses

Mixed model analyses of variance (ANOVAs, SPSS, v24) included factors of Group and repeated measures of Predictability and Intelligibility for the behavioral study and for imaging results in the LH and RH. Per participant dependent variables in the behavioral study were performance accuracy scores and, in the imaging study, average percent signal changes per run for each analyzed cortical parcel. Further, separate ANOVAs analyzed results from parcels in the LH, RH, and comparisons between LH vs. RH with predictability and intelligibility as repeated measures for each group. All analyses of imaging data were for matching parcels. An advantage of averaging percent signal changes within parcels was an improved signal to noise ratio and statistical power without spatial smoothing (Glasser et al., 2016). Added planned comparisons were *t*-tests of group contrasts at each intelligibility level (collapsed across predictability levels) and at each predictability level (collapsed across intelligibility levels). Planned comparisons guarded against missing possible significant group differences for individual intelligibility or predictability levels. We applied Bonferroni adjustments for all analyses (each ANOVA and *post hoc t*-tests). Data for most parcels were normally distributed; non-parametric analyses confirmed findings for parcels with deviated distributions.

## Results

### Behavior Study Findings

A three-way ANOVA found group (*p* ≤ 0.01), intelligibility (*p* ≤ 0.001) and predictability (*p* ≤ 0.001) as significant factors for the task of naming the last word of sentences. Mean last word identification accuracies for 4-, 8-, and 16-bands LP sentences, respectively, in SSD were 9, 51, and 88% and in NH 18, 60, and 89%. Mean accuracies for HP sentences were higher and for the same respective bands were 16, 78, and 97% in SSD and 30, 94, and 100% in NH. Group mean accuracies for 8- and 16-band HP sentences were significantly lower in SSD than NH (Figure 1, red brackets; all *p* values ≤ 0.5). Accuracies in both groups were significantly lower for less intelligible sentences in *post hoc* tests (e.g., 4 vs. 8, 4 vs. 16, and 8 vs. 16) with the sole exception of 8 vs. 16 HP sentences in NH (Figure 1, green brackets; all *p* values ≤ 0.001). Accuracies were significantly lower for LP than HP sentences for 8- and 16-bands in SSD and all bands in NH (Figure 1, blue brackets; all *p* values ≤ 0.002).

### Overview of Imaging Study Findings

**FIGURE 3:**
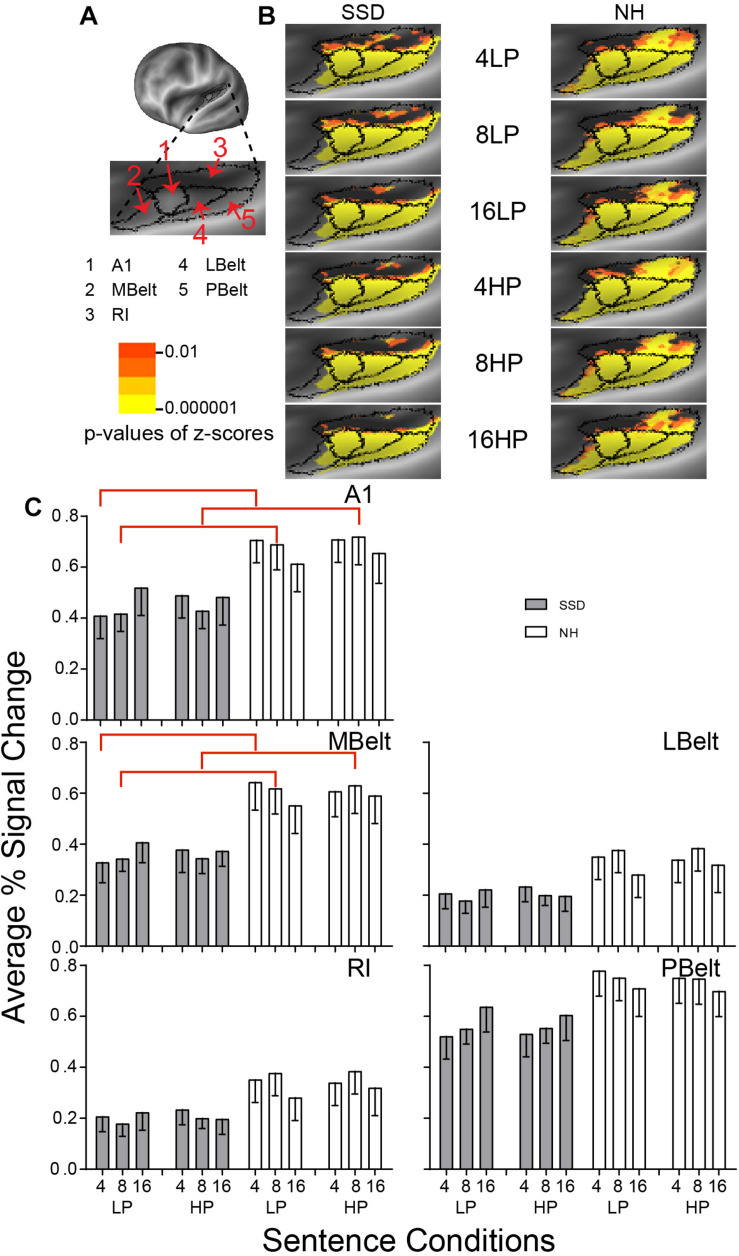
Results from five parcels in the left hemisphere (LH) Early Auditory Cortex (EAC) region. **(A)** A lateral view of an inflated LH reveals the location of EAC and its five parcels along the superior temporal plane. A lateral and inferior rotation revealed the superior temporal plane. **(B)** Enlarged views of parcels show clusters with significant mean z-scores of *p* < 0.01 for positive BOLD (colored red-orange to bright yellow), for each sentence condition (rows) in the SSD and NH groups (left and right columns). Results in the top and bottom three rows were, respectively, for LP and HP sentences. Results displayed in successive three rows per predictability level were for increasingly intelligible sentences with 4-, 8-, and 16-band noise-vocoding. **(C)** Bar graphs show mean and SEM of percent signal change averaged across all vertices per named parcel in LH cortex. A1 and MBelt had significant group differences marked with red brackets. Vertical arrangement of sentence conditions within each panel follows the same sequence shown in B. NH, normal hearing (white bars); SSD, single sided right ear deafness (gray bars).

**FIGURE 4:**
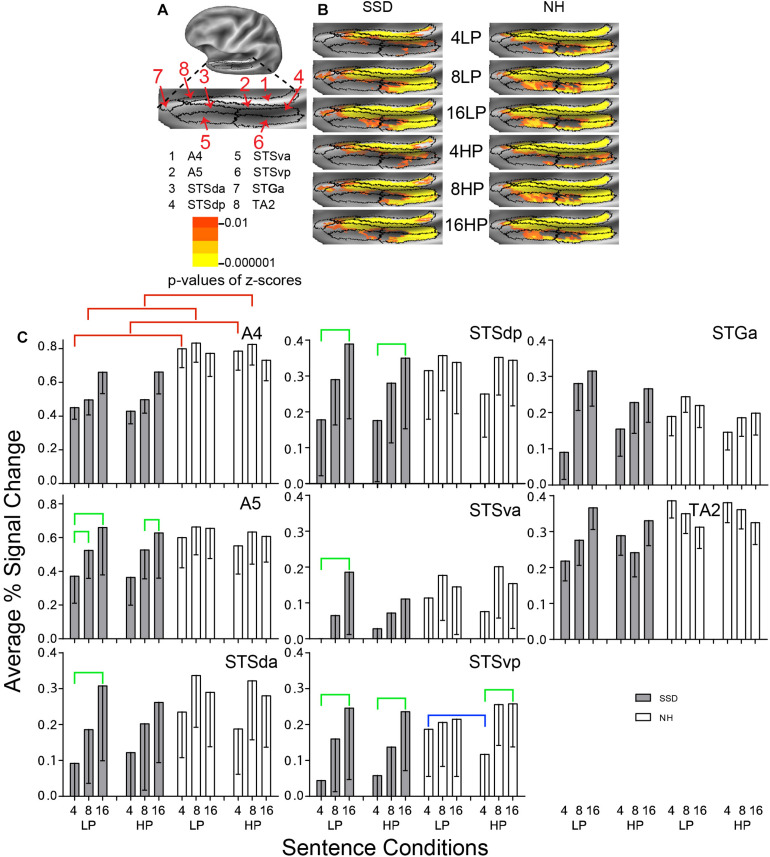
Results from eight parcels in the LH Auditory Association Cortex (AAC) region. **(A)** Lateral view of a partially inflated left hemisphere slightly rotated inferior. **(B)** Enlarged views of parcels with clusters scaled as in Figure 3. **(C)** A4 had significant group effects marked with red brackets. A5, STSda, STSdp, STSva, and STSvp had significant intelligibility effects marked with green brackets. STSvp had a significant predictability effect marked by a blue bracket.

**FIGURE 5:**
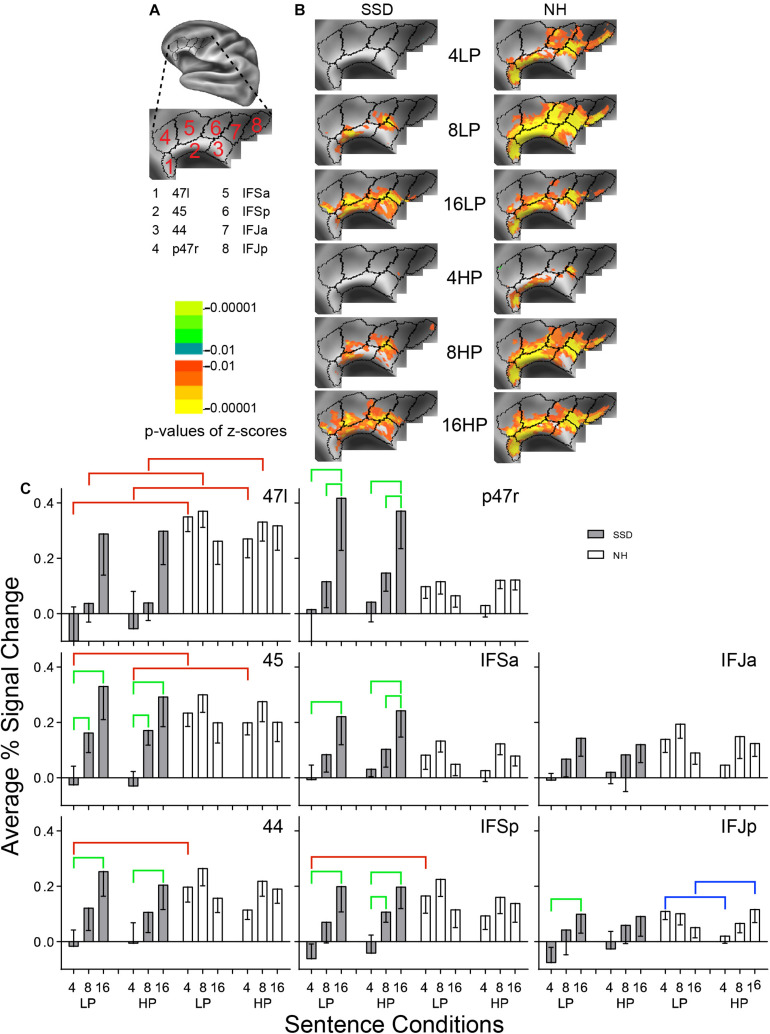
Results from eight parcels in the LH Inferior Frontal Cortex (IFC) region. **(A)** Lateral view of a partially inflated hemisphere rotated superior and tilted slightly medial. **(B)** Enlarged views of parcels with clusters scaled as in Figure 3. **(C)** 47l, 45, 44, and IFSp had significant group effects marked with red brackets. 45, 44, p47r, IFSa, IFSp, and IFJp had significant intelligibility effects marked with green brackets. IFJp had a significant predictability effect marked by a blue bracket.

**FIGURE 6:**
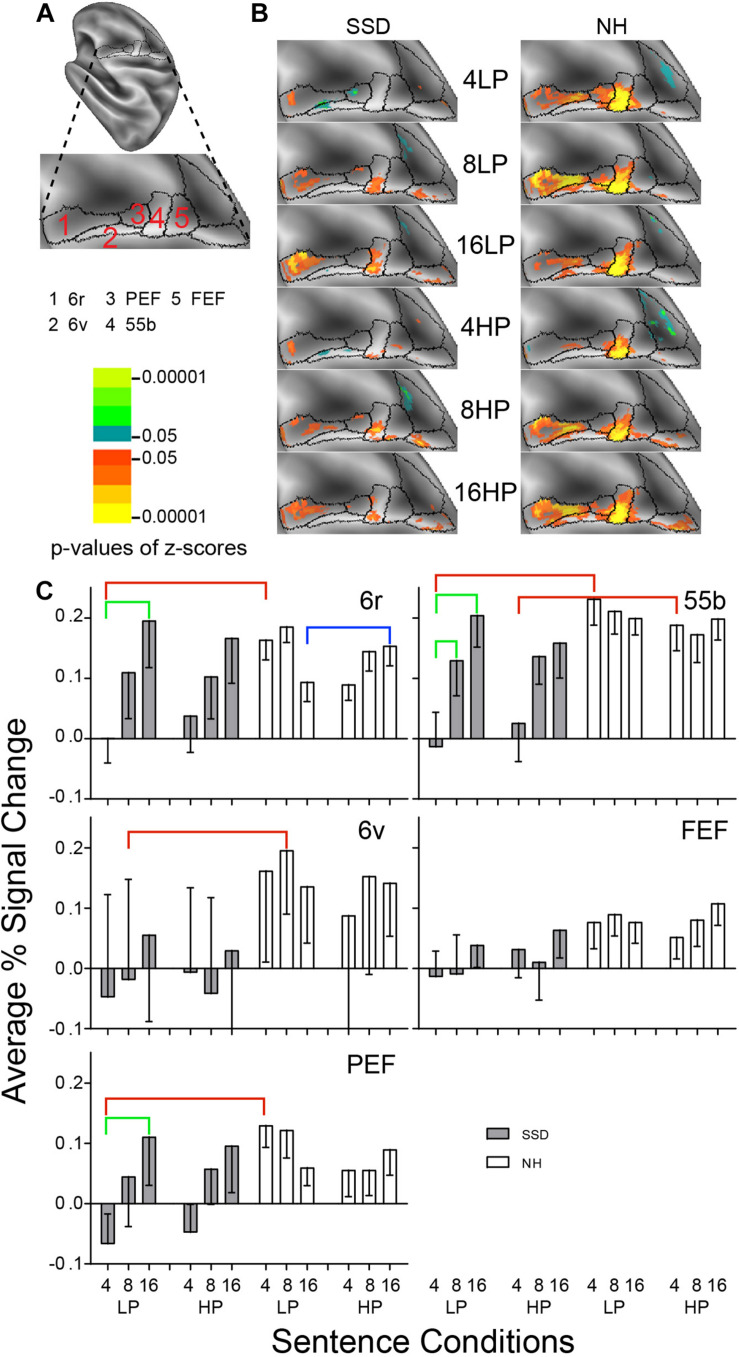
Results from five parcels in the LH Premotor Cortex (PmC) region. **(A)** Lateral view of a partially inflated hemisphere rotated 45 degrees clockwise to align the PmC region horizontally. **(B)** Enlarged views of parcels show clusters with significant mean z-scores of *p* < 0.05 for positive BOLD (colored red-orange to bright yellow) and negative BOLD scaled blue to light green. **(C)** 6r, 6v, PEF, and 55b had significant group effects marked with red brackets. 6r, 6v, and 55b had significant intelligibility effects marked with green brackets. 6r had a significant predictability effect marked by a blue bracket.

**FIGURE 7:**
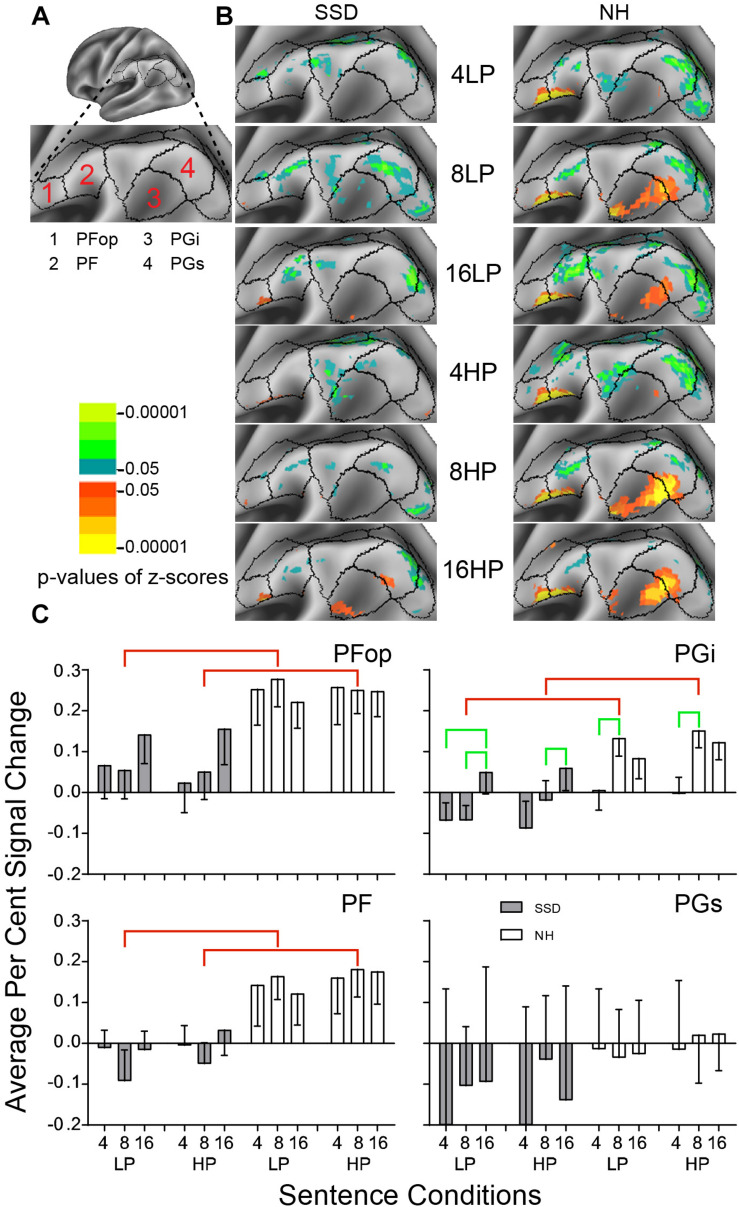
Results from four parcels in the LH Inferior Parietal Cortex (IPC) region. **(A)** Lateral view of a partially inflated hemisphere. **(B)** Enlarged views of parcels show clusters with significant mean z-scores of *p* < 0.05 for positive BOLD (colored red-orange to bright yellow) and negative BOLD scaled blue to light green. **(C)** PFop, PF, and PGi had significant group effects marked with red brackets. PGi had significant intelligibility effects marked with green brackets.

**FIGURE 8:**
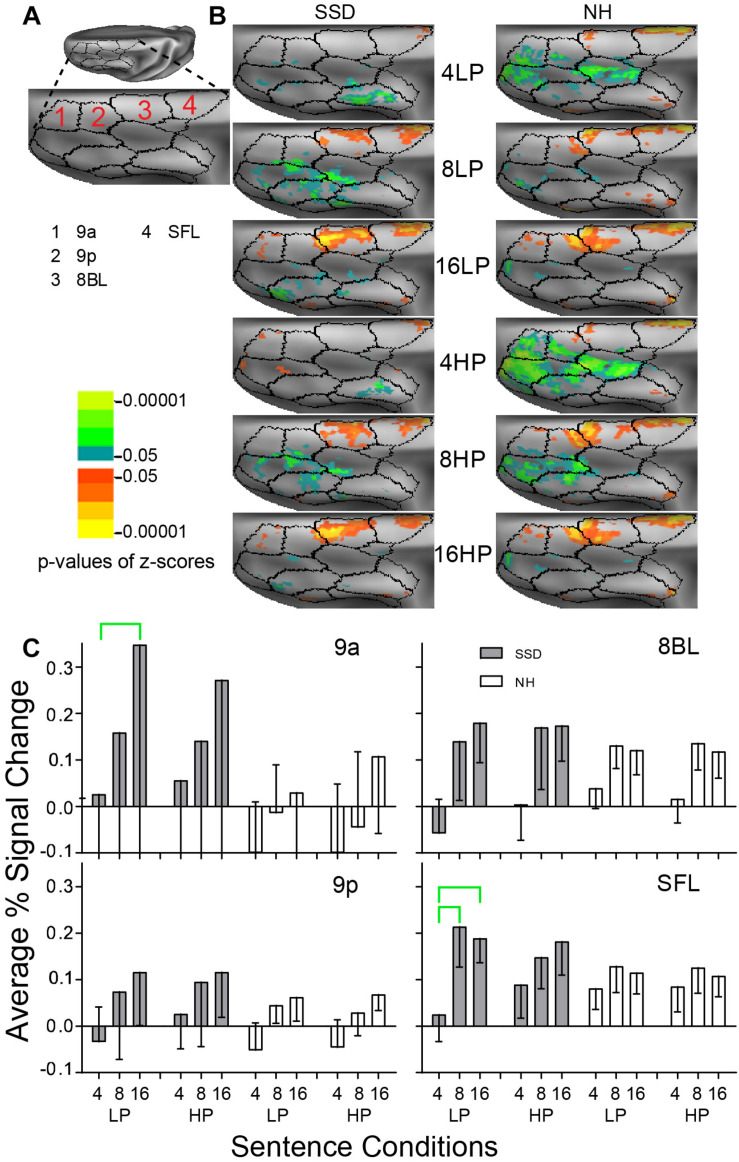
Results from four parcels in the LH Dorsolateral Prefrontal Cortex (DLPFC) region. **(A)** A dorsal view of a partially inflated hemisphere shows the DLPFC region. **(B)** Enlarged views of parcels show clusters with significant mean z-scores of *p* < 0.05 for positive BOLD (colored red-orange to bright yellow) and negative BOLD scaled blue to light green. **(C)** 9a and SFL had significant intelligibility effects marked with green brackets.

**FIGURE 9:**
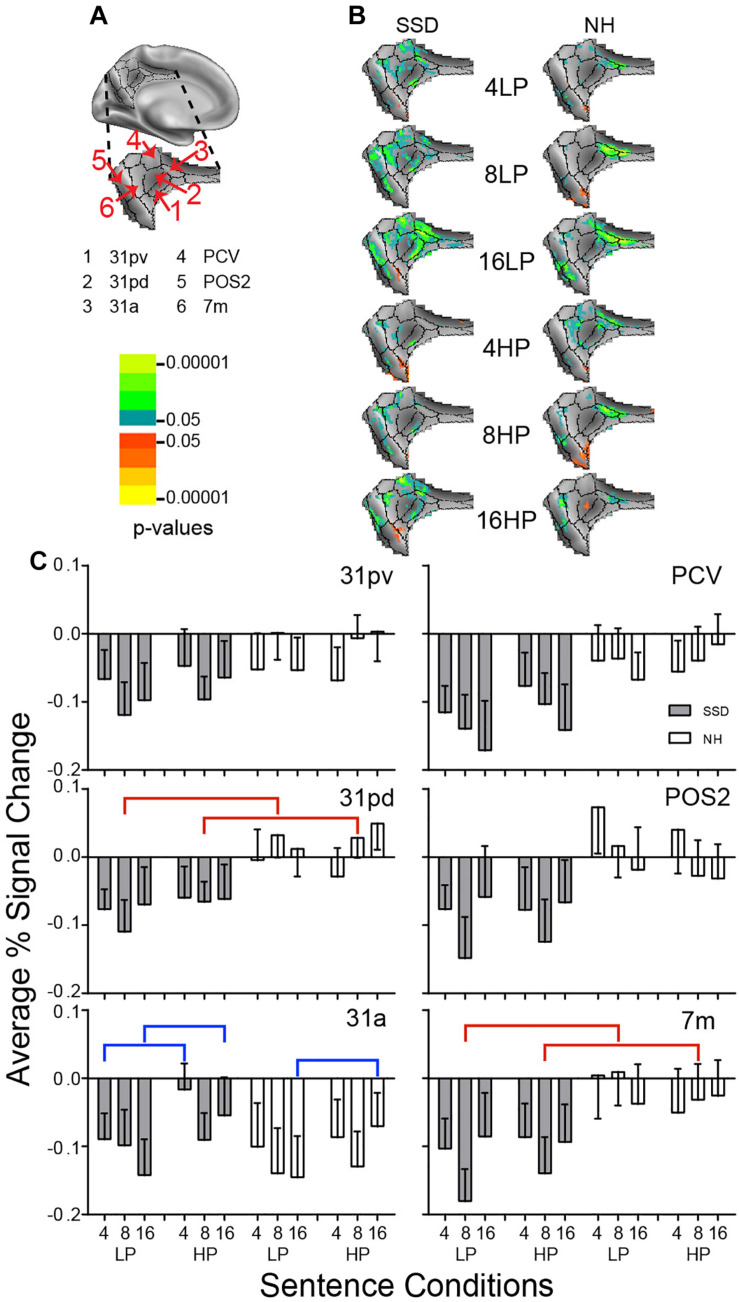
Results from six parcels in the LH Posterior Cingulate Cortex (PCC) region. **(A)** Medial view of a partially inflated hemisphere shows the PCC region. **(B)** Enlarged views of parcels show clusters with significant mean z-scores of *p* < 0.05 for positive BOLD (colored red-orange to bright yellow) and negative BOLD scaled blue to light green. **(C)** 31pd and 7m had significant group effects marked with red brackets. 31a had a significant predictability effect marked by a blue bracket.

**FIGURE 10:**
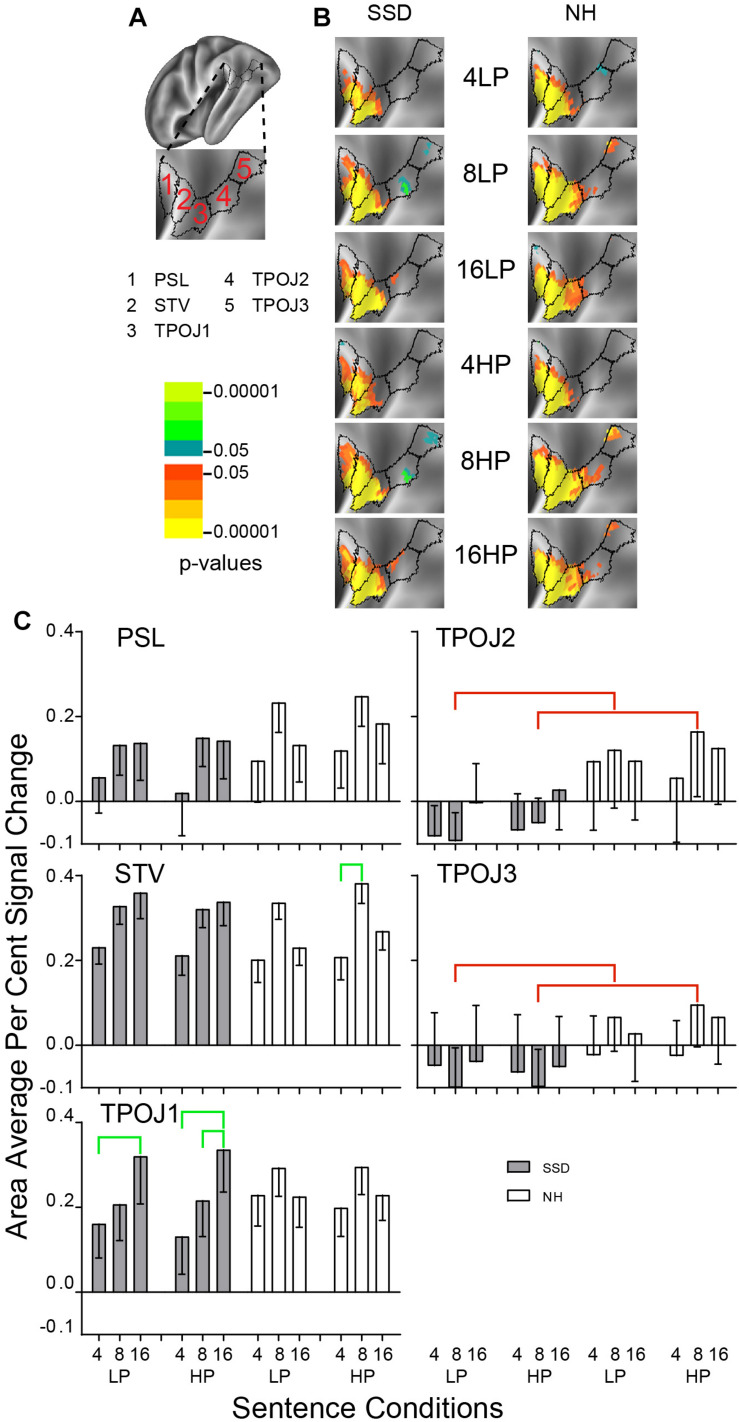
Results from five parcels in the LH Temporal-Parietal-Occipital Junction (TPOJ) region. **(A)** Lateral view of a partially inflated hemisphere rotated superior 45° to align the parcels. **(B)** Enlarged views of parcels show clusters with significant mean z-scores of *p* < 0.05 for positive BOLD (colored red-orange to bright yellow) and negative BOLD scaled blue to light green. **(C)** TPO2 and TPO3 had significant group effects marked with red brackets. STV and TPOJ1 had significant intelligibility effects marked with green brackets.

**FIGURE 11:**
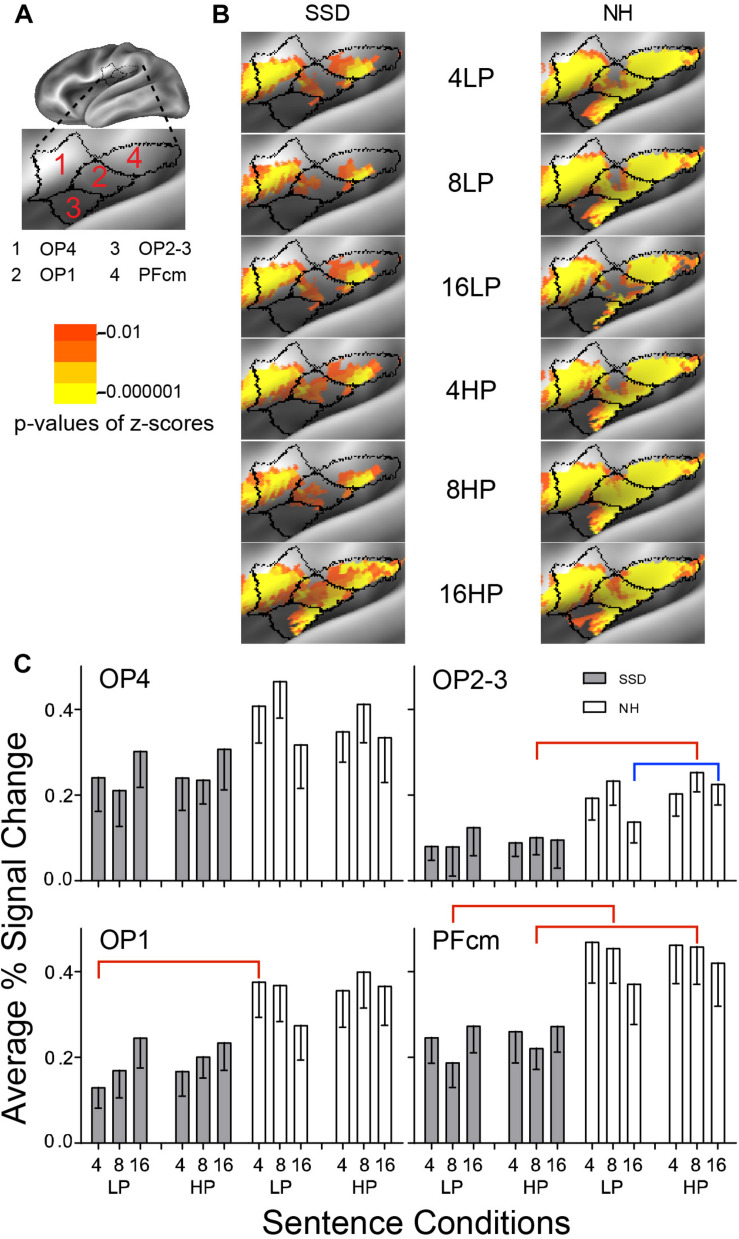
Results from four parcels in the LH Posterior Opercular Cortex (POC) region. **(A)** Lateral view of a partially inflated hemisphere tilted superior 10° to expose the parietal operculum. **(B)** Enlarged views of parcels show clusters with significant mean z-scores of *p* < 0.01 for positive BOLD (colored red-orange to bright yellow). **(C)** OP1, OP2-3, and PFcm had significant group effects marked with red brackets. OP2-3 had a significant predictability effect marked by a blue bracket.

Figure 2 shows an overview of activated cortical locations based on similar maximum activations to 16- and 8-band HP sentences, respectively, in SSD and NH. Activity predominated in multiple left hemisphere regions (Figures 2A,B, LH). Panels B in Figures 3–11 illustrate specific activated LH regions and variations in the extent of activations by different sentence task conditions. The sentence task activations occurred, especially in LH cortical regions in superior temporal gyrus/sulcus (STG/STS), inferior frontal cortex (IFC), inferior parietal cortex (IPC), part of premotor cortex (PmC) in the precentral gyrus (PCG), dorsolateral prefrontal cortex (DLPFC), posterior cingulate cortex (PCC), temporal-parietal-occipital junction, and posterior opercular cortex. Black borders in Figure 2 illustrate a few parcels previously established with multi-modal parcelation criteria unique to every parcel in the cortical regions (Glasser et al., 2016). Figures 3–11 illustrate selected parcels in predominantly activated cortical regions in both groups. Less extensive distributions of activity appeared in similar right hemisphere regions (Figure 2, RH and S2). Black arrows in Figure 2 marks BOLD responses in bilateral medial occipital cortex (centered in primary visual cortex, V1) and left-hand representation in primary somatosensory cortex (S1). These activations possibly reflected, respectively, visual cues and fingertip contact when pressing a response key during each trial.

### Imaging Results by Region

In Figures 3–11, panels A illustrate optimally oriented parcel alignments within a LH cortex region shown on a partially inflated surface. Panels B show significant z-score cluster distributions separately for each sentence condition (4, 8, and 16 band sentences) by group. Top and bottom three rows in each column, respectively, are for low and high predictability (LP and HP) sentences. Panels C show bar graphs of average percent signal changes and standard errors in gray and white bars, respectively, in SSD and NH from named parcels (Glasser et al., 2016) per sentence condition. Text and Supplementary Figures present findings from the RH (Supplementary Figure 2) and differences between hemispheres (Supplementary Figure 3 LH vs. RH).

#### Summary Features

(1) LH parcels showed significant group effects from lower average percent signal changes in SSD than NH for sentences with matched intelligibility and predictability. Multiple regions had parcels with significant group effects: 2 in early auditory cortex (EAC), 1 in auditory association cortex (AAC), 4 in inferior frontal cortex (IFC), 3 in premotor cortex (PmC), 3 in inferior parietal cortex (IPC), 2 in posterior cingulate cortex (PCC), 4 in TPOJ, and 4 in posterior opercular cortex (POC).
(2) Significant intelligibility effects were prevalent in LH parcels from SSD: 5 in AAC, 6 in IFC, 3 in PmC, 1 in IPC, 2 in DLPFC, and 3 in TPOJ; and in NH: 1 in AAC, 1 in IPC, and 1 in TPOJ. Most often significance arose from lower average percent signal changes for 4- than 16-band sentences; less prevalent were lower amplitudes for 4- than 8-band and for 8- than 16-band sentences.
(3) Predictability effects were rare and predominantly part of a significant 3-way interaction (I × P × G). The effect arose from lower amplitudes for LP than HP sentences.
(4) Hemisphere differences arose from significantly higher amplitude responses in LH than RH in matched parcels.

#### Early Auditory Cortex (EAC)

LH. In SSD and NH, each of the 5 EAC parcels had widespread, uniformly distributed significant z-score clusters for all sentence conditions. Z-scores were significant with mostly *p* ≤ 10^–6^ from positive BOLD (Figure 3B, yellow). Group differences were notable in MBelt and RI from a paucity of significant clusters predominantly in ventral/anterior MBelt and dorsal/caudal RI.

Every parcel in EAC had lower average percent signal changes in SSD than NH for nearly every sentence condition (Figure 3C).

Parcel Primary Auditory (A1) had a significant group difference in planned comparisons and Parcel Medial Belt Complex (MBelt) had a significant ANOVA group effect for 4- and 8-band sentences (ps ≤ 0.05). Both parcels had significantly lower amplitudes in SSD than NH for 4-band LP and 8-band LP and HP sentences (Figure 3C, red brackets; all *p* values ≤ 0.05).

RH. Parcel Lateral Belt Complex (LBelt) had a significant ANOVA intelligibility by group interaction effect (*p* ≤ 0.05). In SSD, an intelligibility effect arose from significantly higher amplitudes for 4- than 8-band HP sentences (Supplementary Figure 2 *p* ≤ 0.05). There were no significant effects in NH.

LH vs. RH. Significant hemisphere differences were in parcel LBelt from SSD and parcels A1 and RI from NH (ps ≤ 0.05). In SSD, parcel LBelt had significantly higher amplitudes in RH than LH for every sentence condition (ps ≤ 0.01). In NH, parcels A1 and RI had significantly higher amplitudes in LH than RH for all sentence conditions except 16- band LP in A1 (Supplementary Figure 3 ps ≤ 0.05) and for all sentence conditions in RI (ps ≤ 0.01).

#### Association Auditory Cortex (AAC)

LH. In SSD, all 8 AAC parcels had significant z-score clusters for 16-band sentences, with the fewest in STGa and STSva. The latter two and STSda had fewer or no clusters for less intelligible 8-band sentences. Cluster distributions were scarcest with the least intelligible 4-band sentences, especially STSva, STGa, and STSvp (Figure 4B SSD). In NH, all parcels had significant z-score clusters for each sentence condition, but cluster spatial extents were least for 4-band sentences in STSva and STSvp (Figure 4B NH).

Positive average percent signal changes predominated in AAC parcels. SSD had lower amplitudes than NH for nearly every 4- and 8- but not 16-band sentences (Figure 4C). Parcel STSva amplitudes were lowest in both groups. Parcel A4 had significantly lower response amplitudes in SSD than NH in planned group comparisons for 4- and 8-band sentences (ps ≤ 0.05) and individually for each predictability (Figure 4C, red brackets; all *p* values ≤ 0.05). Differences arose from significantly lower amplitudes in SSD than NH for 4- and 8-band sentences at each predictability level.

Parcels Auditory 5 Complex (A5) and all STS parcels (da, dp, va, and vp) had significant intelligibility ANOVA effects (ps ≤ 0.05). Intelligibility effects occurred exclusively in SSD except STSvp (Figure 4C, green brackets; all *p* values ≤ 0.05). Amplitudes were significantly lower for 4- than 16-band LP sentences in five parcels (A5, STSda, STSdp, STSva, STSvp) and for HP sentences in parcels STSdp and STSvp. Parcel A5 had significantly lower amplitudes for 4- than 8-band LP and for 8- than 16-band HP sentences.

Parcel STSvp in NH had a significant 3-way Intelligibility × Predictability × Group (I × P × G) interaction ANOVA effect (*p* ≤ 0.05). Differences arose from significantly higher amplitudes for 4-band LP than HP sentences in NH (Figure 4C, blue bracket; all *p* values ≤ 0.05).

Anterior temporal pole parcels STGa and TA2 showed no significant sentence condition effects.

RH. Parcel STSdp had a significant 3-way I × P × G interaction ANOVA effect (*p* ≤ 0.05). In SSD, an intelligibility effect arose from significantly lower amplitudes to 4- than 16-band LP sentences; and a predictability effect arose from significantly higher amplitudes to 4-band LP than HP sentences (Supplementary Figure 2 ps ≤ 0.05). In NH, no AAC parcels showed response differences.

LH vs. RH. Significant hemisphere differences were in parcels STSda and STSdp from SSD and NH (ps ≤ 0.05). In SSD and NH, the LH had significantly higher response amplitudes than RH in STSdp, for 8- and 16-band LP and HP sentences (Supplementary Figure 3 ps ≤ 0.05) and mostly similarly in STSda for 8- and 16-band LP and HP sentences (ps ≤ 0.05 not shown).

#### Inferior Frontal Cortex (IFC)

LH. In SSD, parcels 47l, 45, 44, p47r, IFSa, IFSp, and IFJa had significant z-scores for 16-band LP and HP sentences. Fewer parcels (45, 44, IFSa, IFSp, and IFJa) had sparser extents of significant cluster for 8-band sentences. There were no significant clusters for 4-band sentences (Figure 5B SSD). In NH, nearly all parcels had significant z-score clusters for 8- and 16-band sentences. Parcels 47l, 45, 44, IFSp, IFSa, and IFSp had significant clusters for 4-band LP, but not similarly for HP sentences (Figure 5B NH).

In SSD, response amplitudes progressed from negative or near zero for 4- to maximums for 16-band sentences within each predictability level (Figure 5C, gray bars). In NH, response amplitudes were slightly higher for 8-band and lower for 4- and 16-band sentences in most parcels, following a shallow inverted U-shape profile (Figure 5C, white bars).

Parcels 47l, 45, 44, and IFSp had significant group differences in planned comparisons for 4-band sentences (parcel 47l also for 8-band and ANOVA) that arose from significantly lower response amplitudes in SSD than NH for 4-band LP sentences in parcels 47l, 45, 44 and posterior inferior frontal (IFSp), similarly in parcels 47l and 45 for 4-band HP sentences, and in parcel 47l for 8-band sentences in *post hoc* tests (Figure 5C, red brackets; all *p* values ≤ 0.01).

Significant intelligibility ANOVA effects occurred in parcels 45, 44, p47r, IFSa, IFSp, and IFJp (ps ≤ 0.05) and a significant intelligibility by group interaction ANOVA effect for all but IFJa and IFJp (ps ≤ 0.05). Significant intelligibility differences arose only in SSD and occurred comparatively from lower amplitudes to less intelligible sentences of 4- vs. 8- and/or 16-band sentences (Figure 5C, green brackets; all *p* values ≤ 0.05).

Normal hearing showed a significant predictability effect that arose from significantly higher amplitudes for LP than HP 4- and the reverse for 16-band sentences, but only in IFJp (Figure 5C, blue brackets; all *p* values ≤ 0.05).

RH. Parcels p47r, IFSp, and IFJp had significant intelligibility by group interaction ANOVA effects (ps ≤ 0.05). In SSD, an intelligibility effect arose from significantly lower response amplitudes for less intelligible LP 4- than 16-band in p47r, IFSp, IFJp, for 4- than 8-band in IFSP, and for 8- than 16-band in sentences in IFSp (ps ≤ 0.05). Further, parcels p47r, IFSp, IFJa, and IFJp had a significant 3-way (I × P × G) interaction ANOVA effect (ps ≤ 0.05). In SSD, parcel p47r had significantly lower amplitudes to 4-band LP than HP sentences; and parcels IFSp and IFJa had higher amplitudes to 8-band LP than HP sentences (Supplementary Figure 2 ps ≤ 0.05). In NH, parcel IFJp had a higher amplitude (less negative) to 4-band LP than HP.

LH vs. RH. Significant hemisphere differences were in parcels 45 and IFJa from SSD (ps ≤ 0.05). Similar LH response predominance occurred in parcels 47l, 45, 44, p47r, IFSa, and IFJp from NH (ps ≤ 0.01). In SSD, the LH had significantly higher amplitudes than RH for 16-band LP and HP sentences in parcels 45 (Supplementary Figure 3 *p* ≤ 0.05) and similarly for IFJa (*p* ≤ 0.05 not shown). In NH, the LH similarly had significantly higher amplitudes than RH for all sentence conditions in parcel 45 (Supplementary Figure 3 ps ≤ 0.05), and similarly in 44 and IFSa for 8- and 16-band sentences, in parcels 47l and p47r, and for 4- and 16-band sentences in parcel IFJp (ps ≤ 0.05 all not shown). Results from parcel 45 illustrate LH predominance extended across most and often all 6 sentence conditions in NH compared to few or no conditions in SSD (Supplementary Figure 3: 6 vs. 2 black brackets).

#### Premotor Cortex (PmC)

LH. In SSD, parcels 6r and 55b had significant positive z-score clusters for most sentence conditions (Figure 6B SSD). In NH, all PmC parcels had significant z-score clusters for nearly all sentence conditions and the greatest extent was in parcel 55b (Figure 6B NH).

In SSD, all PmC parcels showed negative or near zero responses for 4-band LP and mostly positive percent signal changes for 8- and 16-band sentences (Figure 6C, gray bars). In NH, all parcels showed positive response amplitudes for all sentence conditions (Figure 6C, white bars).

Parcels Rostral Premotor (6r), 55b, Ventral Premotor (6v), and Premotor Eye Field (PEF) had significant group effects (in ANOVA for 6r and planned comparisons otherwise) for 4-band sentences in 55b, 6v and PEF and 8-band sentences in 6r. The effect arose from lower amplitudes in SSD than NH (Figure 6C, red brackets; all *p* values ≤ 0.05).

Parcels 6r, PEF and 55b had significant intelligibility or intelligibility by group interaction ANOVA effects (Figure 6C, green brackets: all *p* values ≤ 0.05). Significant intelligibility differences arose only in SSD from lower amplitudes for 4- than 16-band LP sentences in parcels 6r, 55b and PEF, and for 4- than 8-band sentences in parcel 55b. The response difference between 4- vs. 16-band in SSD for HP sentences was borderline significant.

Parcel 6r had a predictability effect from a three-way I × P × G ANOVA effect in NH, which arose from significantly lower amplitudes for LP than HP 16-band sentences (Figure 6C, blue bracket; all *p* values *p* ≤ 0.05).

RH. Parcels 6r, 6v, and PEF had a significant 3-way I × P × G effect (ps ≤ 0.05). In SSD, parcel PEF had significantly lower negative amplitudes for 4-band LP than HP sentences (*p* ≤ 0.001). In NH, parcel 6r had significantly higher amplitudes for 8- than 16-band LP sentences; and parcel 6v had significantly higher amplitudes for LP than HP 4-band sentences (Supplementary Figure 2 ps ≤ 0.05).

LH vs. RH. SSD showed no significant hemisphere differences. In NH, parcels 6r, 55b and PEF showed significant hemisphere differences (ps ≤ 0.01). In NH, LH parcels 6r and 55b had significantly higher amplitudes than RH for all sentence conditions (Supplementary Figure 3 ps ≤ 0.01) and in PEF for 4-band LP and HP and for 16-band HP sentences (ps ≤ 0.05 not shown).

#### Inferior Parietal Cortex (IPC)

LH. In SSD, parcels Opercular Area PF (PFop) and Inferior Area PG (PGi) had scant positive significant z-score clusters for 16-band sentences. Other parcels had patches of negative z-score clusters (Figure 7B SSD). In NH, parcels PFop, PF, PGi and Superior Area PG (PGs) had significant positive z-score clusters for 8- and 16-band sentences. Parcels PFop and PF had few positive clusters for 4-band sentences (Figure 7B NH). Parcels PF and PGs had negative z-score clusters, less for 16-band HP than other conditions (Figure 7B NH).

In SSD, all parcels except PFop mostly had negative percent signal changes. In NH, all parcels except PGs had positive percent signal changes (Figure 7C).

Parcels PFop, PF and PGi had significant group effects (in ANOVA for PF and planned comparisons otherwise). The effect arose from lower amplitudes in SSD than NH for 8-band sentences when collapsed across predictabilities and for 8-band sentences (Figure 7C, red brackets; all *p* values ≤ 0.05).

Parcel PGi had a significant intelligibility ANOVA effect (*p* ≤ 0.01). In SSD, negative amplitude responses were significantly lower for 4- and/or 8-band than positive amplitudes for 16-band sentences. In NH, positive amplitudes for 4- were significantly lower than for 8-band sentences (Figure 7C, green brackets; all *p* values ≤ 0.05).

RH. Parcels PF, PFop, and PGi had significant 3-way (I × P × G) interaction ANOVA effects (ps ≤ 0.05). In SSD and NH, only parcel PFop had significantly lower amplitudes for 4-band LP than HP, and PFop in SSD also showed significantly higher amplitudes for 16-band LP than HP sentences (Supplementary Figure 2 ps ≤ 0.05). PF and PGi had no further RH specific significant effects.

LH vs. RH. Significant hemisphere differences were in parcel PGi from SSD and parcels PFm, PGi, and PGs from NH (ps ≤ 0.01). In SSD, the RH had significantly lower (more negative) amplitudes than LH in parcel PGi for 8- and 16-band LP and HP sentences (Supplementary Figure 3 ps ≤ 0.05). In NH, the RH had significantly lower (more negative) amplitudes than LH in parcel PGi for all sentence conditions (Supplementary Figure 3 ps ≤ 0.01). Similar amplitude differences were present in PFm for 8- and 16-band and in PGs for 8- HP and 16-band sentences (ps ≤ 0.05 not shown).

#### Dorsolateral Prefrontal Cortex (DLPFC)

LH. In SSD, parcels 9a, 8BL and lateral superior frontal (SFL) had positive z-score clusters for 8- and 16-band sentences (Figure 8B SSD). Cluster extents were larger in 8BL and SFL for 8- and 16-band sentences and scantly present in parcel 9a for 16-band sentences (Figure 8B SSD). In NH, 9a, 9p, 8BL, and SFL had significant positive z-score clusters for 16-band sentences and similarly in 9p, 8BL and SFL for 8-band sentences. The 4-band sentences affected few patches in 8BL and SFL (Figure 8B NH). Negative z-score clusters were absent in the four analyzed parcels in SSD but in NH were in parcels 9a and 9p and elsewhere in DLPFC for 4-band sentences (Figure 8B).

Parcels 9a and SFL had significant intelligibility ANOVA effects (ps ≤ 0.05). The effect in SSD arose from significantly lower positive amplitudes for 4- than 16-band LP sentences in 9a and SFL and for 4- than 8-band sentences in SFL (Figure 8C, green brackets; all *p* values ≤ 0.05).

RH. Parcels 9a and 9p had significant 3-way (I × P × G) interaction ANOVA effects (ps ≤ 0.05). In SSD, parcels 9a and 9p had significantly lower amplitudes for LP than HP 4-band sentences (Supplementary Figure 2 ps ≤ 0.05). In NH, parcel 9a had significantly lower (more negative) amplitudes for LP than positive responses for HP 16-band sentences (Supplementary Figure 2 *p* ≤ 0.05).

LH vs. RH. Significant hemisphere differences were in parcel 8BL from SSD and parcels 8BL and 9p from NH (ps ≤ 0.01). In SSD, the LH had significantly higher amplitudes than RH in parcel 8BL for 8-band HP sentences (Supplementary Figure 3 *p* ≤ 0.05). In NH, the LH had significantly higher amplitudes than RH in parcel 8BL for 8-band and 16-band LP sentences (Supplementary Figure 3 ps ≤ 0.05) and in parcel 9p for 8-band HP sentences (ps < 0.05 not shown).

#### Posterior Cingulate Cortex (PCC)

LH. In SSD, all parcels:(31pv, 31pd, 31a, PCV, POS2, and 7m) had negative BOLD clusters. In NH, all sentence conditions activated negative BOLD clusters in parcel 31a; remaining parcels showed scattered and few negative z-score clusters (Figure 9B NH).

In SSD compared to NH, all parcels except 31a showed greater depth of negative percent signal changes (Figure 9C). Amplitudes of negative depths often were greatest for 8- or 16-band sentences (Figure 9C, gray bars). In NH, parcels other than 31a showed minimal negative responses; parcels 31pd and POS2 had small positive percent signal changes (Figure 9C, white bars).

Parcels 31pd and 7m had significant group effects (in ANOVA for 31pd and planned comparisons in 7 m) that arose from more negative amplitudes in SSD than positive amplitudes in NH for 8-band LP and HP sentences (Figure 9C, red brackets; all *p* values ≤ 0.05).

Parcel 31a had a significant predictability ANOVA effect (*p* ≤ 0.01). Amplitudes were significantly more negative for LP than HP 4- and 16-band sentences in SSD and for 16-band sentences in NH (Figure 9C, blue brackets; all *p* value ≤ 0.05).

RH. No parcel showed a significant effect in either group.

LH vs. RH. Significant hemisphere differences were in parcels 31a, 31pd, and 31pv from SSD and parcels 7m, 31pd, and 31pv from NH (ps ≤ 0.05). In SSD, the LH had significantly less negative (higher) amplitudes than RH in 31pv for 8- and 16-band LP and HP sentences (Supplementary Figure 3 ps ≤ 0.05), similarly in 31pd, and in 31a for 4- and 16-band HP sentences (ps ≤ 0.05 not shown). In NH, the LH had significantly less negative (higher) amplitudes than RH in parcel 31pv for 8-band HP and 16-band LP sentences (Supplementary Figure 3 ps ≤ 0.05), similarly in 31pd for all sentence conditions, and in 7 m for 4-band LP and HP sentences (ps ≤ 0.05 not shown).

#### Temporal-Parietal-Occipital Junction (TPOJ)

LH. In SSD, parcels posterior sylvian language (PSL), superior temporal visual (STV) and TPOJ1 had significant z-score clusters for each sentence condition with the smallest extents for 4-band sentences. Parcels TPOJ2 and TPOJ3 had few positive and small extents for negative clusters, mainly for 8-band sentences. In NH, PSL, STV, and TPOJ1 had extensive significant positive z-score clusters for all sentence conditions. TPOJ2 and TPOJ3 had small significant positive clusters mostly for 8-band sentences.

Parcels TPOJ2 and TPOJ3 had significant group effects in planned comparisons for 8-band sentences collapsed across predictabilities (Figure 10C, red brackets, all *p* values < 0.05). The group effect in both parcels arose from significantly lower amplitude percent signal changes in SSD or each predictability compared to larger amplitudes in NH for HP sentences.

Parcels TPOJ1 in SSD and STV in NH had significant intelligibility ANOVA effects (ps ≤ 0.05). In SSD, an intelligibility effect in TPOJ1 arose from significantly lower positive amplitudes for 4- than 16-band LP and HP and for 8- than 16-band HP sentences. In NH, the intelligibility effect in STV arose from significantly lower amplitudes for 4- than 8-band HP sentences (Figure 10C, green brackets: all *p* values < 0.05).

RH. No parcel showed a significant effect in either group.

LH vs. RH. Significant hemisphere differences were in parcel STV from SSD and parcels STV, TPOJ1, and TPOJ2 from NH (ps ≤ 0.01). In SSD, the LH had significantly higher amplitudes than RH in parcel STV for 8- and 16-band LP and HP sentences (Supplementary Figure 3 ps ≤ 0.01). In NH, the LH had significantly higher amplitudes than RH in parcel STV for nearly all sentence conditions (Supplementary Figure 3 ps ≤ 0.01), nearly the same in TROP1, and in TPOJ2 for 8-band HP and 16-band LP and HP sentences (ps ≤ 0.05 not shown).

#### Parietal Opercular Cortex (POC)

LH. In SSD, parcels OP1, OP4 and PFcm showed extensive significant positive z-score clusters for all sentence conditions; OP2-3 had small patches and only for 16-band sentences. In NH, all parcels showed highly significant widespread clusters for all sentence conditions (Figure 11B, NH).

Parcels OP1, OP2-3, and PFcm had significant group effects in planned comparisons for sentences with 4-band LP in OP1, 8-band HP in OP2-3, and 8-band LP and HP in PFcm, collapsed across predictabilities (ps ≤ 0.05). Group differences arose from significantly lower percent signal changes in SSD than NH (Figure 11C, red brackets; all *p* values ≤ 0.05).

In NH, parcel OP2-3 had a significant predictability effect for 16-band HP sentences in planned comparisons (<0.05). The predictability effect arose from significantly lower amplitudes for LP than HP sentences (*p* = 0.004).

RH. Parcels OP1 and OP2-3 had a significant group effect when collapsed across predictabilities in planned comparisons (ps ≤ 0.05). Group differences arose from significantly lower amplitudes in SSD than NH for 8-band LP and HP sentences in OP1 (Supplementary Figure 2, red brackets; all *p* values ≤ 0.05) and similarly in OP2-3 (ps ≤ 0.05 not shown).

Parcels OP1, OP4, and PFcm also had significant 3-way (I × P × G) interaction Anova effects (ps ≤ 0.05). Predictability differences arose from significantly lower amplitudes for LP than HP 16-band sentences in OP1 (Supplementary Figure 2, blue brackets; all *p* values ≤ 0.05) and similarly in PFcm (*p* ≤ 0.05 not shown).

LH vs. RH. There were no significant hemisphere differences in parcels from SSD. Such differences occurred in parcel PFm from NH (ps ≤ 0.01). In NH, the LH had significantly higher amplitudes than RH in parcel PFcm for all sentence conditions (Supplementary Figure 3 ps ≤ 0.01).

## Discussion

### Behavior Study

Single sided deafness had lower performance accuracies than NH in naming sentence last words for all sentence conditions, despite no significant group differences in age or hearing between the better ear in SSD and each ear in NH participants. These group differences were significant for HP 8- and 16-band but not LP or 4-band HP sentences. NH also obtained greater benefit than SSD from moderately and more intelligible, predictable sentences. In contrast, accuracies were worse in both groups for less intelligible sentences.

Right ear deaf SSD led to significantly reduced accuracies in recognizing target words in spectrally degraded vocoded sentences. Possibly, inferior performance arose from right ear deafness causing deficient activation of left early auditory cortex, important for processing speech acoustics (Zatorre et al., 2002). Yet, diminished processing of speech acoustics might not fully account for reduced accuracy in semantic processing of spectrally degraded speech. Besides, intact left ear inputs expanded activation of left early auditory cortex in right ear SSD (Burton et al., 2012). Further, diminished neural activity in other cortical regions might contribute to speech recognition difficulties in SSD.

Sentence intelligibility and semantic predictability similarly affected accuracies in both groups, which were lowest for the least intelligible low semantic predictability (LP) sentences. Accuracies for the least intelligible 4-band HP sentences were also low, showing HP did not compensate for the least intelligible sentences. These findings for 4-band sentences suggest limits to top-down influences on semantic processing. Conversely, accuracies in both groups were excellent for the most intelligible 16-band sentences, irrespective of sentence predictability, although SSD had lower scores than NH. Both groups were more correct with high than low predictability of moderately intelligible 8-band sentences. Accessible sentence syntax in HP sentences may have aided interpreting moderately intelligible sentences. Previously, training individuals to attend phonemes and phonology improved accuracies with spectrally degraded speech (Davis et al., 2005). Prior training to attend speech acoustics could direct a top-down focus and might be beneficial for SSD individuals, especially for understanding speech in noise.

### Imaging Study: General Effects

#### Group Differences

Response amplitudes were significantly lower in SSD than NH to sentences presented with 4- and 8-bands, respectively, in 15 and 32 LH parcels. No parcels showed group amplitude differences with 16-band sentences. A further > 70 LH parcels showed non-significant differences ranging from 0.1 to 0.25% lower signal amplitudes in SSD than NH for 4- and/or 8-band sentences. In SSD, possibly even small signal declines affected recognition of speech distorted by adverse noise vocoded spectral degradation.

No parcels from the RH showed an ANOVA effect for group (see Supplementary Figure 2). There also was no evidence of enhanced, compensatory RH activity in SSD despite acoustic inputs exclusively from the left ear.

#### Intelligibility Effects

In SSD, parcels in multiple cortical regions had significantly less activation to sentences that were less understandable due to fewer frequency bands after noise vocoding. Examples were significantly lower response amplitudes to less intelligible 4- or 8- compared to 16-band sentences. Speech may become less comprehensible because of depressed activation in multiple components of a cortical language network. Activity reductions may result with speech distortions caused by noise vocoded spectral degradation or unfavorable environmental noise. Unknown is whether both speech distortion methods reduce brain activity similarly. Spectral degradation effects in SSD were more prevalent for LP than HP sentences (25 vs. 17 instances). Reduced semantic predictability may also have lowered response amplitudes and adversely affected sentence intelligibility.

RH parcels rarely showed an intelligibility effect, confirming earlier findings in NH (Scott et al., 2000; Narain et al., 2003). Six RH parcels from three cortical regions (EAC, AAC, and IFC) in SSD and two (EAC and IFC) in NH showed an intelligibility effect. Response amplitude differences for less intelligible sentence bandwidths were inconstantly lower or higher. Generally, RH effects were suspect due to the rarity and absent pattern of response amplitude differences. Prior studies found pitch variation activated RH STG/STS cortex (Scott et al., 2006; Obleser et al., 2008; Woods et al., 2011; Kyong et al., 2014; Plack et al., 2014). The latter indirectly concurs with finding no significant intelligibility effects in RH parcels in both groups.

#### Predictability Effects

Predictability levels significantly influenced brain activity as a contributing factor, mainly in 3-way interactions. Contrasts between responses to LP and HP sentences were rarely significant in *post hoc* tests. Significantly higher response amplitudes for LP than HP sentences occurred in the LH for one parcel in SSD, 3 in NH, In the RH, 3 parcels in SSD and 2 in NH had higher amplitudes to LP sentences. Significantly lower amplitudes for LP than HP occurred in the LH for 1 parcel in NH. In the RH, 4 parcels in SSD and 2 in NH had lower amplitudes to LP sentences. The findings of predictability effects were inadequate for further analysis or comparison with findings from the Behavioral Study.

### Specific Findings by Cortical Region

#### Early Auditory Cortex (EAC)

Single sided deafness had lower response amplitudes than NH for all sentence conditions in all EAC parcels. Group differences were significant in parcels A1 and MBelt. SSD likely had diminished, but not eliminated, processing of basic acoustic features because of deficient response amplitudes in nearly all EAC parcels. Consequently, auditory association cortex possibly received impoverished signals.

Both groups showed no significant intelligibility effects in EAC parcels. Thus, SSD and NH had relatively comparable percent signal change amplitudes for all sentence conditions (Figure 3C). Therefore, any deficient processing of speech acoustics in EAC probably did not solely cause sentence intelligibility problems in SSD.

A prior study considered lower response amplitudes from speech distortions and noise-vocoding of word lists as an intelligibility effect in EAC belt and parabelt areas of NH participants (Davis and Johnsrude, 2003). However, assessed participant accuracies signified number of remembered or recollected individual words from a word list after a delay (Davis and Johnsrude, 2003). The tested recall in the prior study did not engage semantics and syntax. The current study used semantics and syntax in a sentence to predict and help name target last words.

#### Auditory Association Cortex (AAC)

Accessory auditory cortex parcels showed intelligibility effects, but only response amplitudes in SSD were significantly lower for less intelligible sentences, particularly 4- vs. 16-band vocoding. Accented speech can also be less intelligible. Prior studies reported accented speech resulted in lower response amplitudes in STG/STS cortex (Davis and Johnsrude, 2003; Adank et al., 2012), which included the AAC region. Accents may alter the phonology of speech, possibly resulting from language affected pitch alterations (Binder et al., 2009; Woods et al., 2011). Consequently, accents might degrade phoneme and phonological coding in SSD, resembling an effect of spectral degradation.

Prior reviews that assessed the role of STG/STS cortex in semantic processing for speech comprehension questioned this role (Binder et al., 2009; Peelle, 2012). However, strokes affecting STG/STS disrupted phonemic and phonological processing, thereby indirectly affecting language semantics (Bogen and Bogen, 1976). In SSD, we found evidence of semantic processing in the behavioral study based on significantly greater than chance accuracies for identifications of sentence last words, despite 8- and 16-band spectral degradation. Possibly, the source for semantic processing difficulties shown by SSD in the behavioral study (and during speech recognition in adverse sound environments) was not exclusive to AAC parcels despite intelligibility effects found in AAC parcels. The latter coincides with concerns expressed in Binder’s meta-analysis of a role for AAC in semantic processing. Our finding of significant intelligibility effects in SSD suggest speech understanding problems reflect more than changes induced by deafness in processing speech acoustics in early auditory cortex.

#### Inferior Frontal Cortex (IFC)

Inferior frontal cortex parcels in SSD showed linear increases in response amplitudes with sentence intelligibility. Spatial extents of significant clusters similarly increased from mostly few for 4- to greatest spatial cluster extents for 16-band sentences. Deficient cognitive effort in semantic processing might have caused lower activation amplitudes and smaller cluster extents for 4- and 8-band sentences in SSD (Badre et al., 2005). Greatest response amplitudes and activation extents to 16-band sentences with SSD, however, might have reflected greater cognitive effort for speech comprehension compared with NH. Sentences with fewer spectral bands possibly exposed deficient semantic processing, which might have resulted from impoverished phonological decoding. In SSD, semantic deficits might have resulted in unrecognized words and consequent missed syntactic linkages. Previous studies found that missing information could degrade use of IFC in retrievals/selections from semantic memory (Badre et al., 2005; Binder and Desai, 2011).

Nearly every IFC parcel in SSD showed a significant sentence intelligibility effect of diminished activation to sentences presented with a less intelligible bandpass. All three inferior strata parcels (47l, 45, and 44) and superior parcel IFSp showed significantly lower response amplitudes in SSD compared to NH for 4-band and 47l also for 8-band. A more challenging speech intelligibility task might have shown corresponding lower activities in other IFC parcels with significantly lower response amplitudes to spectrally degraded speech.

Inferior frontal cortex and IPC are particularly important in semantic processing when retrieving from a stored lexicon (Badre and Wagner, 2002; Scott et al., 2006; Obleser et al., 2007b; Hervais-Adelman et al., 2012). SSD might then poorly detect word and sentence meaning in IFC and IPC (Turken and Dronkers, 2012). However, there were no significant group performance differences for 4-band sentences despite significant group activation differences; and there were significant performance differences for 16-band sentences but no significant group activation differences. Hence, IFC might not be solely responsible for performance accuracies in the behavioral study because of no correspondence between significant group differences in performance accuracies and activation magnitudes for all sentence intelligibilities.

Higher positive amplitude responses uniquely occurred in parcel p47r from SSD to 16-band sentences. Previously, bilateral activation occurred in this anterior superior parcel from NH adults in contrasts between paired objects identified by feature dimensions and verbal categories [Supplementary Neuroanatomical Results in Glasser et al. (2016)]. The prior task required multilevel semantic definitions of objects, based on physical dimensions, and for words, based on categorization, to match relational vs. categorical objects. The current sentence task similarly compelled semantic searches for meaning of sentence words and syntactic links between them to identify target last sentence words. Higher amplitudes in p47r of SSD may show more extensive word searches of related verbal classes in the spoken sentences. Hearing deficits in SSD possibly impoverished word clarity from inadequate phonological decoding, which may have needed extra retrieval tries from a stored lexicon to match and understand speech, hence greater activation.

#### Premotor Cortex (PmC)

Previously, a language relatedness effect activated parcel 55b when NH participants matched verbal object categories and identified relationships between interacting vs. random objects [Supplementary Neuroanatomical Results in Glasser et al. (2016)]. The sentence task required making associations between spoken words as a predictor of sentence last words. Relatedness in semantic links between words in sentences might have thus activated parcel 55b. Similarly, SSD vs. NH showed no clusters for 4- and sparse, lower significance clusters for 8- and 16-band sentences. SSD individuals possibly sought links between sentence words only for 8- and 16-band sentences because these had greater clarity. In contrast, NH could have detected intelligibility in all sentences, leading to extensive searches for relationships between words. NH coincidently showed highly significant clusters irrespective of sentence clarity. Less activity in 55b of SSD than NH suggests greater difficulty connecting word meanings across speech, thereby possibly limiting recognition of semantic relatedness.

Usage of working memory to review spectrally degraded speech might explain increased activity in parcel 6r. Previously, parcel 6r in NH adults responded during a working memory task, when listening to a story, answering arithmetic questions or matching objects to a verbal category [Supplementary Neuroanatomical Results in Glasser et al. (2016)]. Parcel 6r response amplitudes and spatial extent of significant clusters were least for 4-band in SSD and similar in both groups for 8- and 16-band sentences. Possibly, both groups exercised working memory during even slightly intelligible speech to recollect a sentence when predicting the last word and NH might have exerted this effort to reconstruct 4-band sentences.

Activation in premotor eye-movement control parcels PEF and FEF was greater in NH than SSD. Activated sectors of PEF and FEF adjoined larger cluster extents in 55b. Potentially there was incorrect assignment of activity to PEF and FEF, which was in 55b. Adjoining border locations between these three parcels might have blurred alignments (Glasser et al., 2016).

#### Inferior Parietal Cortex (IPC)

In SSD, activity in IPC parcels PGi/PGs was slight and only for highly predictable 16-band sentences. Less intelligible sentences activated negative BOLD responses in these parcels. In NH, PGi/PGs showed response amplitudes near zero for 4-, greatest for 8- and slightly lower for 16-band sentences. A comparable inverted U-shaped profile in NH previously followed the effort level needed to encode and/or retrieve semantics from speech (Tune and Asaridou, 2016), suggesting NH exerted greater effort for moderately intelligible speech. Prior studies in NH adults also reported activation in the left AG to spectrally degraded speech (Davis and Johnsrude, 2003; Peelle et al., 2010; Obleser and Kotz, 2011; Hervais-Adelman et al., 2012; Golestani et al., 2013; Seghier, 2013) and particularly to verbal working memory of recent speech (Obleser et al., 2007a; Seghier, 2013). These prior findings suggest, weak activation of PGi in SSD might show diminished ability to retrieve sentence relevant information. Diminished retrospective retrievals may show less integration of degraded speech by SSD individuals to decide a global sentence meaning (Humphries et al., 2006).

#### Dorsolateral Prefrontal Cortex (DLPFC)

Working memory (WM) contributes to comprehending degraded speech through retrospective reconstruction of missed syntax, words, and phonemes (Mattys et al., 2012). Prior studies in NH found activation of DLPFC for tasks requiring “maintenance and manipulation of information within working memory” [p. 139 in Kim et al. (2012)]. In SSD, WM might not have successfully aided retrospective sentence or word reconstructions. Yet, exercise of executive processes for retrieval may have improved chances of recollection (Spaniol et al., 2009), resulting in finding enhanced activation of some parcels in DLPFC of SSD. Hence, efforts to retrieve degraded speech with a degree of intelligibility (e.g., 8- and 16-band noise-vocoding) could explain the higher amplitude responses found in DLPFC parcels of SSD. Positive response amplitudes to 8- and 16-band sentences were higher in SSD than NH, notable in parcels 9a and 8BL. Group differences in parcel 9a were significant. Higher response amplitudes in DLPFC parcels in SSD also may reflect hearing deficits, which made it difficult to fully understand spectrally degraded sentences.

#### Posterior Cingulate Cortex (PCC)

Both groups showed negative BOLD responses in PCC during the sentence task. Depth of negative BOLD was greater in SSD. The activity pattern in PCC likely reflected the role of this region as a hub for the “resting” state, default mode network (DMN) (Fox et al., 2005; Dosenbach et al., 2007; Raichle and Snyder, 2007; Buckner et al., 2008). Prior studies reported negative BOLD in the DMN when attending non-personal, external events/tasks (Raichle and Snyder, 2007). In contrast, self-referential episodic behavior resulted in lesser negative BOLD magnitudes (Buckner and Carroll, 2007). Naming the last word in sentences demanded attention, and especially so with spectral degradation. Spectrally degraded spoken sentences were more difficult to understand with SSD, potentially requiring greater effortful attention to external events. Hence, greater negative BOLD amplitudes in PCC of SSD suggest these individuals had reduced resting states. Greater negative BOLD in PCC might show more effort by the SSD group to attend speech because of hearing deficits.

Significant negative BOLD signals also occurred in IPC parcels PFt, PFm, and PGp. Amplitudes were more negative in SSD than NH, a likely result because these parcels functionally connect with DMN and show more negative signal amplitudes during tasks compared to rest (Fox et al., 2005; Raichle and Snyder, 2007).

#### Temporal-Parietal-Occipital Junction (TPOJ)

Left hemisphere parcels in TPOJ showed significantly lower response amplitudes in SSD than NH for the sentence task. Previously, activation of RH TPOJ involved tasks with multiple targets and cues to disengage a current focus of attention (Corbetta and Shulman, 1998). The sentence task did not involve selective cuing of spoken words or directed attention to a different task/object (Mattys et al., 2012). Consequently, significant group differences in parcels from TPOJ in SSD unlikely resulted from changes in attention. Besides, attention effects previously predominated in the right TPOJ, thus inconsistent with current LH findings. Significant intelligibility effects noted in STV of NH and TPOJ1 of SSD suggested lower activation to less intelligible speech. Again, these findings did not suggest shifts in attention involving speech. A future cued language task might examine the effects of attention in SSD.

#### Posterior Opercular Cortex (POC)

Previously, evoked activity occurred in POC to electrical stimulation of the cochlea in animals (Woolsey and Walzl, 1982), sounds in humans (Eickhoff et al., 2006), somatosensory stimulation in animals and humans (Eickhoff et al., 2007; Burton et al., 2008) and currently to attended speech. Activation also occurred in POC during articulation (Guenther et al., 2006; Seghier et al., 2015), suggesting a possible basis for prior evidence of multimodal activation. Connections between POC and motor cortex might involve a feedback role during speech linked to somatosensory and auditory inputs. Unexpected physical perturbations of somatosensory feedback during speech evoked negative BOLD in POC and during error prone object naming that affected articulation, especially bilaterally in area OP1 (Seghier et al., 2015). Larger amplitude positive BOLD occurred, however, during articulation in motor cortex face/head musculature regions partly linked to speaking (Golfinopoulos et al., 2011). The presence of only positive BOLD in POC bilaterally in both groups during the sentence task might show absence of overt articulation. However, non-overt articulatory rehearsal might aid recollection of spectrally degraded sentences. Minimal non-explicit articulatory rehearsal due to poor speech recollection in SSD may explain significantly smaller response amplitudes to the sentences in this group compared to NH. A possible consequence of smaller amplitudes response in POC might be absent non-overt articulatory rehearsals in SSD to poorly understood spectrally degraded sentences.

#### Left vs. Right Hemisphere Contrasts

Left hemisphere parcels showed significantly higher amplitude responses in both groups. RH parcels showed no evidence of compensatory enhanced speech processing in right ear deaf. Parcel LBelt from EAC in the SSD group was unique in showing significantly lower amplitude LH responses for all sentence intelligibilities. Greater response amplitudes in the LH, despite lost right ear inputs, possibly arose through ipsilateral connections from the intact left ear (Bilecen et al., 2000; Burton et al., 2012). Likely contributing to LH dominance were commissural cortical and/or subcortical bilateral connections, especially below the inferior colliculus (Langers et al., 2005). However, there was a different pattern in SSD compared to NH among parcels with significant LH vs. RH differences. The pertinence of these group differences in patterns of LH dominance are dubious considering the task design was not ideal for examining hemisphere differences between paired interhemispheric parcels.

### Study Limitations

The study included a small heterogeneous sample of participants whose demographic backgrounds varied (e.g., etiology). Age at onset of SSD, particularly congenital versus non-congenital onset, and duration of deafness are important variables in the development of auditory pathways (Gordon et al., 2010; Tibbetts et al., 2011; Kral et al., 2013a,b; Tillein et al., 2016; Polonenko et al., 2018). The majority of participants had adult onset of SSD; however, one lost hearing at age 6 years and two had assumed congenital onset. Degree of residual hearing in the affected ear is another critical factor in auditory development (Sininger et al., 2010; Davidson et al., 2014, 2019; Tomblin et al., 2015). Eight participants had no auditory thresholds at the limits of the audiometric equipment, and four had hearing thresholds in the severe to profound range (including the two with assumed congenital onset). It is possible that even limited amounts of hearing contribute to auditory processing and cortical activation patterns. Additional study with a homogenous and larger SSD group is needed to determine the extent of these factors on study findings.

Participants pressed a key during imaging to show whether they predicted sentence target words. Key presses avoided inhalation shifts to speak, which would have altered BOLD signals from changes in vascular concentrations of CO_2_. However, key presses during the imaging study did not identity target words, nullifying accuracy assessments.

Differences in predictability effects between the behavioral and imaging studies possibly arose from sentence presentation protocols. For the behavioral study, we randomized presentation order of sentence conditions. During imaging, predictability level was random within a block of 10 successive sentences, but every sentence in a block had the same noise-vocoded bandwidth. Participants could have ignored sentence predictability with the block design and attended primarily to sentence intelligibility, thus minimizing attention to syntactic predictability.

FreeSurfer (Dale et al., 1999) generated cortical surface data from each participant, enabling subsequent registration of each hemisphere to a standardized template (Van Essen et al., 2012). Registration matrices also helped projection of average parcel borders onto prior average left and right cortical surfaces (Glasser and Van Essen, 2011; Van Essen et al., 2012). Consequently, we examined parcel specific activity differences between groups within predefined borders, optimally established independent of current imaging results (Kriegeskorte et al., 2009). The approach accurately found where groups differed. Parcel borders, however, might not have precisely aligned in every brain. Determining parcel borders in individual brains would have been optimal [Supplementary Methods in Glasser et al. (2016)]. Available structural MR image sequences were inadequate for creating individualized parcel borders as in the Human Connectome Project. Spatial smoothing potentially obscured topographical patterns and networks of functional/anatomical organization [Supplementary Results and Discussion, p20 in Glasser et al. (2016)]. Fortunately, all participant brains were “typical” since surface reconstructions showed every studied brain with parcel 55b nestled between FEF medially and PEF laterally in the premotor cortex region, a cortex region typically prone to misaligned parcels (Glasser et al., 2016).

Parcels lacking statistical normality in findings from the vertices might have affected estimated average activation from a parcel. Factors potentially responsible for absence of normality might have been lower signal change per vertex for less intelligible or predictable sentence conditions. However, data analyzed with parametric and non-parametric tests found identical significant effects. Consequently, we elected to use parametric test results as these tend to offer greater robustness.

## Conclusion

Both groups showed comparable activation to the most intelligible 16-band sentences. Nearly matched performance accuracies in both groups for 16-band sentences further suggested SSD retained semantic processing capabilities like possibly those in NH. Deficits from SSD, however, probably involved widespread reductions in activity throughout components of a domain general language network. Smaller amplitude responses found in left EAC of SSD does not fully account for speech recognition deficiencies from right ear deafness. Degraded acoustic signal processing in EAC parcels probably precipitated weaker phonemic and phonological processing in AAC parcels. Subsequently affected parcels receiving diminished phonemic signals in semantic control regions chiefly involved reduced activation in inferior strata parcels in the inferior frontal cortex, parcel PGi in the angular gyrus in anterior inferior parietal cortex, and processing relatedness between words in parcel 55b of premotor cortex. Transmission of weakened signals throughout the speech network potentially hindered retrievals from a lexicon. Hence, SSD participants understood spectrally degraded sentences poorly, an effect like an adverse sound environment. However, the same LH brain locations processed speech semantics despite right ear deafness. Predominant group differences involved significantly smaller response amplitudes in SSD than NH groups, especially to spectrally 8-band degraded speech in LH parcels. There was no evidence of compensatory semantic processing in RH parcels from SSD participants. Significant intelligibility effects resulted from lower response amplitudes to 4- compared to 8- and 16-band degraded sentences. Intelligibility effects occurred in all studied regions except early auditory, posterior cingulate cortex and posterior opercular cortex. Parcels with intelligibility effects predominated in the SSD group, mainly resulting from lower amplitude responses to sentence with greater spectral degradation. Intelligibility effects predominated in language network regions found in selected parcels from auditory association, inferior frontal, inferior parietal, and premotor cortex. The sentence task minimally involved differences between SSD and NH participants in domain cognitive control regions. Exceptions included greater negative BOLD in default mode regions of SSD, possibly from greater attention to the sentences. Greater effort by SSD to retrieve sentence syntax retroactively may also have led to higher response amplitudes in DLPFC parcels.

## Data Availability Statement

The raw data supporting the conclusions of this article will be made available by the authors, without undue reservation.

## Ethics Statement

The studies involving human participants were reviewed and approved by Human Research Protection Office. The patients/participants provided their written informed consent to participate in this study.

## Author Contributions

HB, JF, and RR designed the study. HB, AA, and RR analyzed the data. HB, AA, and TH collected imaging data. JF and RR recruited participants and collected behavior data. HB wrote the manuscript with assist from JF and RR. All authors contributed to the article and approved the submitted version.

## Conflict of Interest

The authors declare that the research was conducted in the absence of any commercial or financial relationships that could be construed as a potential conflict of interest.

## Footnotes

1 http://lcni.uoregon.edu/~jolinda/MRIConvert/

2 http://www.fmrib.ox.ac.uk/fsl/

3 http://brainvis.wustl.edu/wiki/index.php/Caret:Operations/Freesurfer_to_fs_LR

4 http://mumford.bol.ucla.edu/

## Supplementary Material

Supplementary Figure 1 Sample interrupted imaging sequence shows 5 trials with 9 s frame repetitions (TR). Each TR starts with a 2 s volume acquisition (TA) followed by a 7 s interval of silence with no pulse sequence noise. Tan and red colored rectangles during scanner silence mark, respectively, presentations of low and high predictability (LP or HP) sentences. The midpoint of each sentence duration, averaging 1.78 s, occurred at the 2 s middle of the sentence presentation rectangles, which aligned 5 s prior to the midpoint of each subsequent TA. Vertical tick marks below the pulse sequence are in 1 s intervals.

Supplementary Figure 2 Bar graphs show mean and SEM of percent signal change averaged across all vertices per named parcel in RH cortex.

Supplementary Figure 3 Bar graphs show mean and SEM of percent signal change averaged across all vertices in matched named parcels in LH and RH. Black brackets mark LH vs. RH significant response amplitude differences of all *p* values < 0.05.
