# Supplementary figures and images for: Cortical Regions Activated by Spectrally Degraded Speech in Adults With Single Sided Deafness or Bilateral Normal Hearing
Source: Front Neurosci. 2021 Apr 7;15:618326. doi: 10.3389/fnins.2021.618326 (PMC8058229; doi:10.3389/fnins.2021.618326)

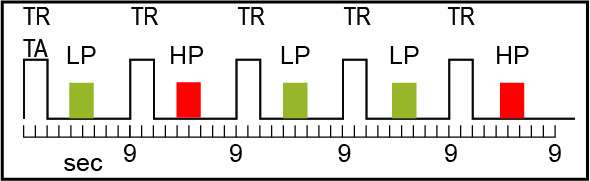

Supplement: Supplementary Figure 1 — Sample interrupted imaging sequence shows 5 trials with 9 s frame repetitions (TR). Each TR starts with a 2 s volume acquisition (TA) followed by a 7 s interval of silence with no pulse sequence noise. Tan and red colored rectangles during scanner silence mark, respectively, presentations of low and high predictability (LP or HP) sentences. The midpoint of each sentence duration, averaging 1.78 s, occurred at the 2 s middle of the sentence presentation rectangles, which aligned 5 s prior to the midpoint of each subsequent TA. Vertical tick marks below the pulse sequence are in 1 s intervals. [file Image_1.jpg]

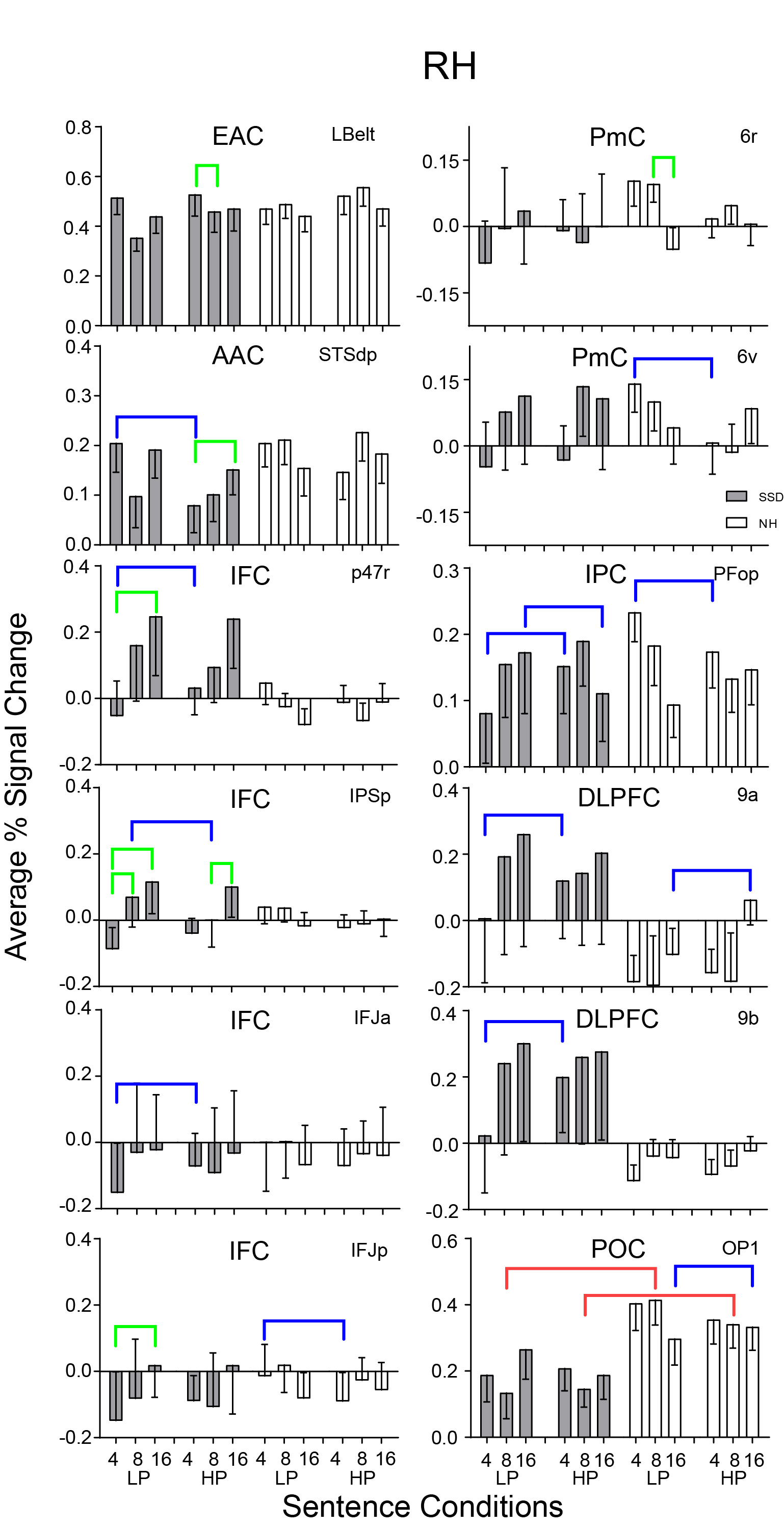

Supplement: Supplementary Figure 2 — Bar graphs show mean and SEM of percent signal change averaged across all vertices per named parcel in RH cortex. [file Image_2.jpg]

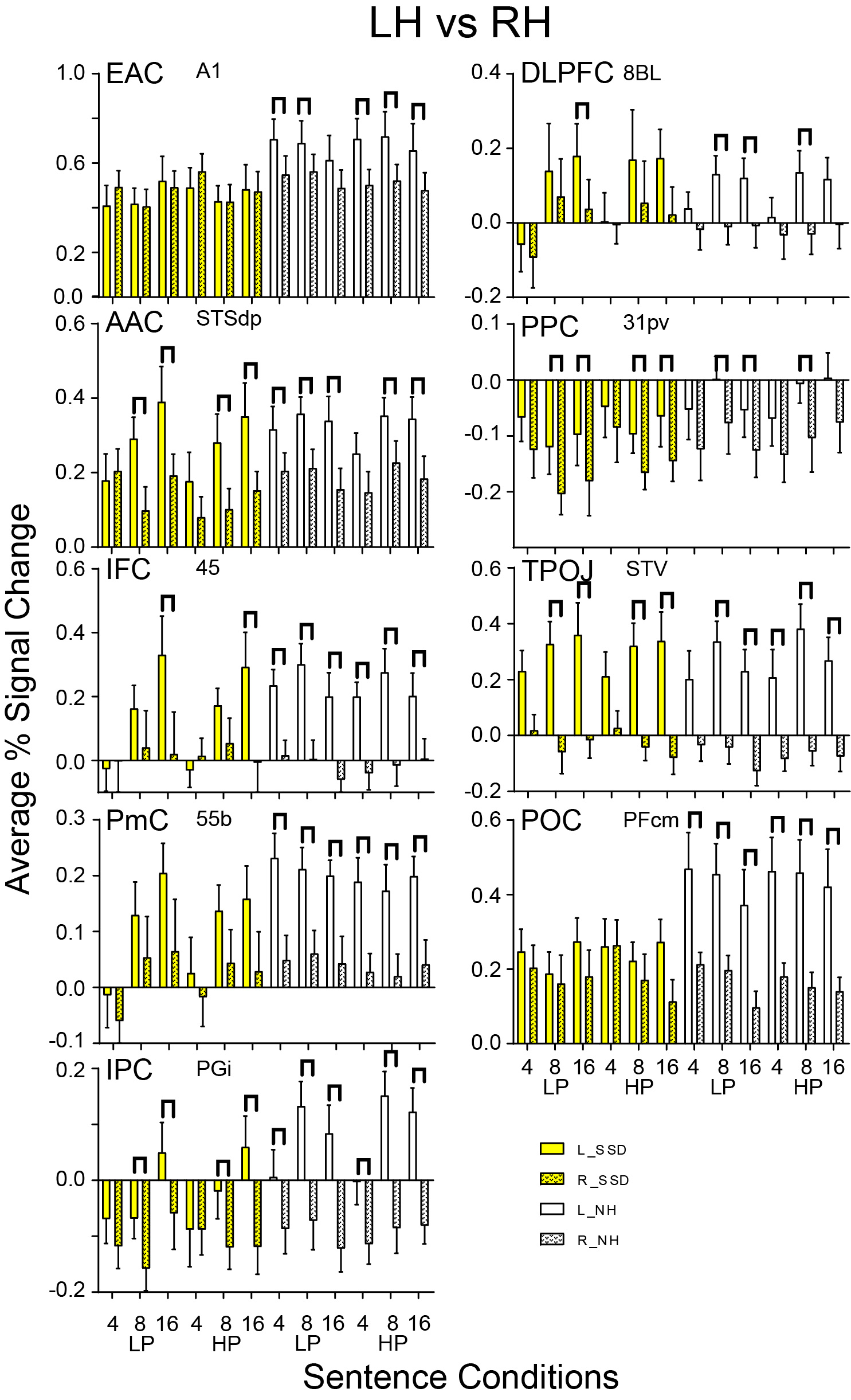

Supplement: Supplementary Figure 3 — Bar graphs show mean and SEM of percent signal change averaged across all vertices in matched named parcels in LH and RH. Black brackets mark LH vs. RH significant response amplitude differences of all p values < 0.05. [file Image_3.jpg]
